# Hybrid deep learning and YAMNet features for asthma diagnosis from respiratory sounds

**DOI:** 10.1038/s41598-026-49247-y

**Published:** 2026-04-29

**Authors:** Ghada Abd El-Latif Shatat, Hossam El-Din Moustafa, Mohamed Sabry Saraya, Al-Saeed Ahmed Marzouk, Asmaa A. Hekal

**Affiliations:** 1 Electronics and Communications Engineering (ECE) Department, Faculty of Engineering, Mansoura University, Mansoura, 35516 Egypt; 2 Computer and Control Systems Engineering, Faculty of Engineering, Mansoura University, Mansoura, 35516 Egypt; 3 Faculty of Computers and Information Systems, Egyptian Chinese University (ECU), Cairo, 11765 Egypt

**Keywords:** Asthma diagnosis, Respiratory sound analysis, Hybrid deep learning, YAMNet embeddings, acoustic feature fusion, Explainable artificial intelligence, Mobile health applications, Biomarkers, Computational biology and bioinformatics, Diseases, Engineering, Health care, Mathematics and computing, Medical research

## Abstract

A prompt and precise diagnosis is necessary to lessen the worldwide burden of asthma, a common chronic respiratory condition. Spirometry is one of the resource-intensive conventional methods that frequently misclassifies cases because of overlapping symptoms with other respiratory illnesses. The Asthma Detection Dataset v2, which is openly accessible and comprises actual audio recordings of respiratory noises, is used in this work to demonstrate a hybrid deep learning architecture for automated asthma detection. After applying thorough preprocessing, which includes denoising, segmentation, and augmentation, the suggested pipeline employs a dual-path feature extraction technique that combines pretrained YAMNet embeddings with handmade acoustic descriptors (MFCCs, chromagram, ZCR, and spectral features). Atrous Spatial Pyramid Pooling (ASPP) and Squeeze-and-Excitation (SE) modules are used to further improve these embeddings, and a Multi-Layer Perceptron is used for classification. The proposed model achieved high performance, with an accuracy of 98.6% ± 0.14, an F1-score of 97.5% ± 0.14, and a macro-AUC of 99.1% ± 0.14 using stratified 5-fold cross-validation. SHAP-based interpretability and visualization (waveforms, spectrograms, t-SNE) verified that the model records clinically significant auditory biomarkers such as crackles and wheezes. These results indicate the potential of the proposed model as an explainable and scalable computer-aided asthma diagnostic tool.

## Introduction

Asthma is a chronic respiratory disease characterized by airway inflammation and variable airflow obstruction, affecting over 262 million people globally and causing approximately 461,000 deaths annually^1^. Common symptoms such as wheezing, coughing, chest tightness, and shortness of breath significantly impair daily activities and quality of life^2^. Although asthma commonly manifests in childhood, it may persist or recur at any age, underscoring the need for timely and accurate diagnosis^3^.

Conventional diagnostic approaches, including spirometry and bronchial provocation testing, remain the clinical gold standards. However, these methods are time-consuming, require specialized equipment, and may not fully capture the heterogeneity of asthma phenotypes^4^. Misdiagnosis is common, as asthma symptoms often overlap with other respiratory conditions such as chronic obstructive pulmonary disease (COPD)^5^. Moreover, asthma arises from complex interactions between intrinsic factors (e.g., genetic predisposition, allergy history) and extrinsic factors (e.g., environmental allergens, pollutants, lifestyle)^6^. Understanding these triggers is essential for precise diagnosis and effective treatment^7^.

The limitations of traditional diagnostic methods highlight the urgent need for innovative, non-invasive, and scalable solutions^8^. Advances in machine learning (ML) and artificial intelligence (AI) have enabled the extraction of hidden patterns from large biomedical datasets, offering personalized, real-time insights into disease detection^9^. Among emerging techniques, respiratory sound-based diagnostics have gained attention as a cost-effective, non-invasive alternative. Deep learning (DL) models can automatically analyze wheezes, crackles, and other acoustic biomarkers to distinguish asthmatic from healthy breathing patterns^10^. Nevertheless, previous research faces major challenges, including small and heterogeneous datasets, inconsistent data collection protocols, lack of standardized evaluation metrics, and limited interpretability^11^.

Asthma is inherently heterogeneous, encompassing multiple phenotypes such as allergic, non-allergic, exercise-induced, and occupational asthma^12,13^. Additional subtypes, including cough-variant asthma, severe asthma, and asthma-COPD overlap syndrome (ACOS), exhibit distinct triggers, severities, and therapeutic responses^14–17^. This diversity underscores the need for precise, phenotype-aware diagnostic frameworks.

To address these challenges, a computer-aided diagnostic (CAD) framework for asthma detection based on hybrid deep learning and explainable AI techniques is proposed. The framework integrates spectrogram-based models with handcrafted acoustic features to enhance diagnostic accuracy, reduce human error, and facilitate real-time telemedicine applications^18^. The main contributions of this work are summarized as follows:

- A dual-path feature extraction mechanism is employed, in which pretrained YAMNet embeddings are combined with handcrafted acoustic features (MFCC, chromagram, ZCR, and spectral descriptors) to capture both interpretable auditory cues and fine-grained temporal-spectral patterns.
- Atrous Spatial Pyramid Pooling (ASPP) and Squeeze-and-Excitation (SE) modules are incorporated to enhance feature representations and improve classification performance.
- The proposed model is evaluated on the publicly available Asthma Detection Dataset v2, where high performance is achieved, with 98.6% accuracy, 97.5% F1-score, and 99.1% macro-AUC under stratified 5-fold cross-validation.
- SHAP-based interpretability techniques are utilized to provide explainable insights into model predictions, enabling clinicians to identify the most influential acoustic features in the diagnostic process.

The remainder of this paper is organized as follows: “Literature review” reviews related work on asthma detection and respiratory sound analysis. “Materials and methods” describes the dataset, preprocessing, feature extraction, and model architecture. “Methodology” presents the proposed hybrid model, while “Results” reports experimental results and comparative analysis. Finally, “Conclusion and future work” concludes the paper and outlines directions for future research.

## Literature review

With the growing interest in audio-based diagnostic systems, the Asthma Detection Dataset v2 has become a pivotal benchmark for respiratory sound analysis. The review begins with the foundational study by Tawfik et al.^19^, which introduced the dataset and established one of the first end-to-end pipelines for asthma detection. The dataset included recordings from clinical and public sources such as ICBHI, with features like Mel-Frequency Cepstral Coefficients (MFCC) and Zero-Crossing Rate (ZCR) extracted alongside spectrograms^19^. The authors used transfer learning to test a DenseNet-based CNN with conventional machine learning models (SVM, Random Forest) for classification; the CNN performed best on spectrogram inputs. The relatively limited dataset ( 1000 samples), lack of specific performance measurements, lack of interpretability techniques, and ignored variability in recording settings were some of the study’s shortcomings despite its contributions. However, it established a baseline for AI-based asthma detection, proving viability while indicating the need for development in terms of scalability, transparency, and standardized assessment.

Kuntalp et al. investigated the incorporation of deep learning methods into Internet of Things (IoT) healthcare applications in their systematic study. The paper cited studies that applied deep learning to biomedical inputs like audio, such as the Tawfik et al. dataset, even though it was not specifically focused on asthma or respiratory audio analysis^20^. The authors organized previous research based on neural network designs, data modalities, and application domains using a structured PRISMA technique. The review is useful for contextualizing remote diagnostics and real-time monitoring because of its extensive coverage of many data types, such as EHR, sensors, images, and audio. However, its immediate significance is diminished by its lack of experimental modeling and its lack of specific attention to respiratory sounds or asthma detection. Nevertheless, the study supports the increasing trend of real-time, lightweight, IoT-driven healthcare solutions, which is in line with the objective of implementing asthma detection models on low-resource or mobile platforms.

Bolhasani et al. examined the quality and character of respiratory sound datasets that were previously available, including the Tawfik et al. dataset^21^. The authors emphasized persistent issues that lower the generalizability and dependability of machine learning systems, such as inconsistent labeling of respiratory problems, background noise, and the lack of uniform collecting techniques, rather than putting forth models. Despite the lack of experimental results or methodological solutions, the study offered important context by highlighting data quality, annotation standards, and transparency in dataset compilation. These findings support the current study’s conclusion that thorough preprocessing, denoising, and quality control are essential when getting the Kaggle dataset ready for deep learning applications.

Hwang et al. used structured environmental and allergy-related data instead of respiratory audio in their investigation to offer a prediction algorithm for diagnosing asthma^22^. Then, they used factors like pollen exposure, dust, pet contact, and allergy history, and then trained models using Random Forest (RF) and Logistic Regression (LR) on a partially overlapping version of the Kaggle dataset. They used SMOTETomek hybrid resampling to correct class imbalance, and the results showed that pollen exposure was the most significant predictor and that RF clearly outperformed LR. The study’s dependence on self-reported tabular data restricts precision and ignores dynamic physiological cues present in respiratory sounds, even while it emphasizes the diagnostic importance of environmental and allergic factors. Its application is further limited by the lack of real-time monitoring and audio analysis. However, it highlights the possibility of combining non-audio characteristics with respiratory sound analysis, bolstering the argument for multimodal models that enhance diagnostic reliability and practical applicability.

A regression tree-based asthma prediction model was presented by Li et al. and improved using the Entropy-Based Subset Selection (E-SS) algorithm^23^. The accuracy of the model was 91.3%, higher than that of Bayesian networks (83.3%). This method’s interoperability is one of its main advantages since it allows doctors to see and comprehend decision processes, which deep learning models frequently do not. However, it was unclear whether the Asthma Detection Dataset v2 was used because the dataset source was not made explicit. Furthermore, the strategy differs fundamentally from audio-driven systems and is limited in its applicability to real-time monitoring because the data were only structured (numerical and categorical) and lacked audio elements. Nevertheless, the study emphasizes the need to integrate interpretability with feature selection, providing insights that could guide the development of audio-based models using methods like decision-path explanations or SHAP values.

Using the SPRSound dataset, which consists of more than 9000 annotated respiratory episodes, Ehtesham et al. created a system for detecting juvenile asthma^24^. They created 512-dimensional embeddings from 2-s audio recordings using Google’s HeAR model after classifying them using several different algorithms. The greatest results were obtained by the MLP (93% accuracy, F1-score 0.88, AUC 0.97), while the generalization of the Logistic Regression was good. The study is appropriate for telemedicine and edge deployment due to its strengths, which include the use of a strong pretrained model, high-quality pediatric data, and the incorporation of interpretability tools like waveform review and PCA visualization. Limitations include limited transparency of HeAR embeddings, limitations to pediatric patients, and confusion between acoustically similar sounds. All things considered, the work offers a sophisticated and practically applicable framework for audio-based asthma identification. The reviewed research that used the Asthma Detection Dataset v2 and related datasets is structuredly summarized in Table 1. It provides a comparative perspective that enhances the narrative debate by outlining the datasets used, study scope, data type, applicable methodology, performance measures, benefits, and limits.

Roy and Satija^25^ proposed AsthmaSCELNet, a lightweight supervised contrastive embedding learning framework for asthma classification using lung sounds. Their approach extracts Mel-spectrogram representations and learns discriminative embeddings through supervised contrastive learning, followed by a multilayer perceptron (MLP) classifier. The proposed model demonstrated strong performance on respiratory sound datasets, highlighting the effectiveness of contrastive learning in capturing subtle acoustic biomarkers such as wheezes and crackles. However, the framework primarily focuses on embedding learning and does not leverage pretrained audio representation models such as YAMNet.

Similarly, Lee et al.^26^ proposed AsTFSONN, a unified framework based on a Time–Frequency Domain Self-Operational Neural Network for asthmatic lung sound classification. The framework exploits joint time–frequency representations of respiratory audio signals in order to capture both temporal breathing dynamics and spectral characteristics associated with pathological respiratory events such as wheezes and crackles. In particular, the proposed self-operational neural network architecture extends conventional convolutional operations by enabling adaptive nonlinear transformations within the network, allowing it to model complex acoustic patterns more effectively than standard deep learning architectures. The model processes time–frequency representations derived from respiratory sound recordings and learns discriminative features that improve the separation between asthmatic and non-asthmatic breathing patterns. Experimental evaluation demonstrated promising classification performance, highlighting the capability of self-operational neural networks to capture subtle acoustic variations in lung sounds. However, the proposed approach mainly relies on handcrafted time–frequency representations and does not exploit transfer learning from large-scale pretrained audio models. Consequently, while the framework demonstrates strong discriminative ability, its generalization capability may be limited when compared to approaches that leverage pretrained audio embeddings such as YAMNet.

Recent studies have investigated the use of pretrained audio neural networks for respiratory sound analysis. For instance, Roy et al.^27^ proposed a mel-spectrogram snippet representation learning framework for detecting the severity of Chronic Obstructive Pulmonary Disease (COPD). Their method utilizes deep audio representations extracted from mel-spectrogram snippets to capture subtle acoustic patterns associated with pathological respiratory events. The experimental results demonstrated the effectiveness of learned audio embeddings in identifying disease-related acoustic characteristics, highlighting the potential of pretrained audio models for respiratory disease assessment and monitoring.

Similarly, Roy et al.^28^ investigated the effect of auscultation hindering noises on the detection of adventitious respiratory sounds using pretrained audio neural networks. Their study evaluated the robustness of models such as YAMNet under different noisy auscultation conditions, highlighting the effectiveness of pretrained audio models for respiratory sound classification tasks in real-world environments.

Recent studies have explored hybrid and ensemble-based learning strategies for biomedical signal analysis and disease diagnosis. For instance, Al-Tam et al.^29^ proposed a hybrid framework that combines a Transformer encoder with residual convolutional neural networks to model both global temporal dependencies and local spectral patterns in heart sound signals for cardiovascular disease recognition. Similarly, Al-Tam et al.^30^ introduced a stacking ensemble-based machine learning approach for Parkinson’s disease diagnosis, where multiple base classifiers trained on tabular clinical features are integrated through a meta-learner to improve predictive accuracy and robustness.

These studies highlight the growing importance of hybrid and ensemble learning paradigms for medical diagnosis tasks. However, they primarily focus on heart sound or tabular clinical datasets. In contrast, the proposed framework in this study specifically targets respiratory sound analysis and employs pretrained YAMNet embeddings combined with Atrous Spatial Pyramid Pooling (ASPP) and Squeeze-and-Excitation (SE) modules to capture multi-scale acoustic representations relevant to asthma detection.

Recent studies have explored several pretrained audio neural networks for respiratory sound analysis, including VGGish, OpenL3, and YAMNet. VGGish and OpenL3 are widely used for extracting generic audio embeddings trained on large-scale datasets such as AudioSet and environmental sound corpora. These models provide robust high-level representations for various audio classification tasks. However, they are typically designed for general-purpose acoustic event detection and may not explicitly capture the fine-grained temporal–spectral patterns present in respiratory sounds.

In contrast, YAMNet offers a lightweight architecture based on MobileNet and provides efficient log-Mel spectrogram embeddings optimized for real-time audio analysis. Previous studies, such as Roy et al. [R4], evaluated the robustness of pretrained audio models under auscultation noise conditions, demonstrating that pretrained embeddings can effectively capture clinically relevant respiratory sound patterns. Motivated by these findings, the proposed framework adopts YAMNet as the base feature extractor and further enhances the learned representations using ASPP and SE modules to capture multi-scale respiratory acoustic patterns.

**Table 1:** Summary of recent research on asthma detection and respiratory sound analysis.

Authors	Year	Methodology	Dataset	Advantages	Limitations
Tawfik et al.	2021	End-to-end asthma detection pipeline using MFCC, ZCR and spectrograms with CNN models	Asthma Detection Dataset v2	Introduced benchmark dataset and baseline models	Limited dataset size and lack of interpretability
Bolhasani et al.	2021	Analysis of respiratory sound datasets focusing on data quality and labeling issues	Various respiratory datasets	Highlighted dataset challenges in respiratory sound analysis	Did not propose a classification model
Kuntalp et al.	2023	Systematic review of deep learning applications in IoT healthcare using PRISMA methodology	Multiple biomedical datasets	Comprehensive overview of AI in healthcare monitoring	No experimental respiratory sound analysis
Hwang et al.	2023	Asthma prediction using Random Forest and Logistic Regression with environmental factors	Kaggle asthma dataset	Considers environmental and allergy features	Does not analyze respiratory audio signals
Roy and Satija	2023	AsthmaSCELNet framework using supervised contrastive learning with mel-spectrogram features	Respiratory lung sound datasets	Lightweight embedding learning for lung sound classification	Does not use pretrained models such as YAMNet
Lee et al.	2023	Time–Frequency Domain Self-Operational Neural Network for lung sound classification	Respiratory lung sound dataset	Captures temporal and spectral respiratory patterns effectively	Relies mainly on handcrafted time–frequency representations
Ehtesham et al.	2025	Asthma detection using pretrained embeddings and multiple ML classifiers	SPRSound dataset	Strong performance using pretrained audio features	Focused on pediatric dataset only
Li et al.	2025	Regression Tree model with Entropy-Based Subset Selection for asthma prediction	Structured asthma dataset	High interpretability for clinical decision-making	Limited to structured data without audio signals
Roy et al.	2025	Study on robustness of pretrained audio neural networks under auscultation noise	Respiratory sound dataset with noise	Evaluates robustness of models like YAMNet in noisy conditions	Focused on noise robustness rather than asthma diagnosis
Roy et al.	2025	Effect of Auscultation Hindering Noises on Detection of Adventitious Respiratory Sounds Using Pre-trained Audio Neural Networks	Respiratory sound dataset with simulated noise conditions	Investigates robustness of pretrained models in realistic auscultation environments	Focused on noise robustness rather than asthma diagnosis specifically

## Materials and methods

**Fig. 1:**
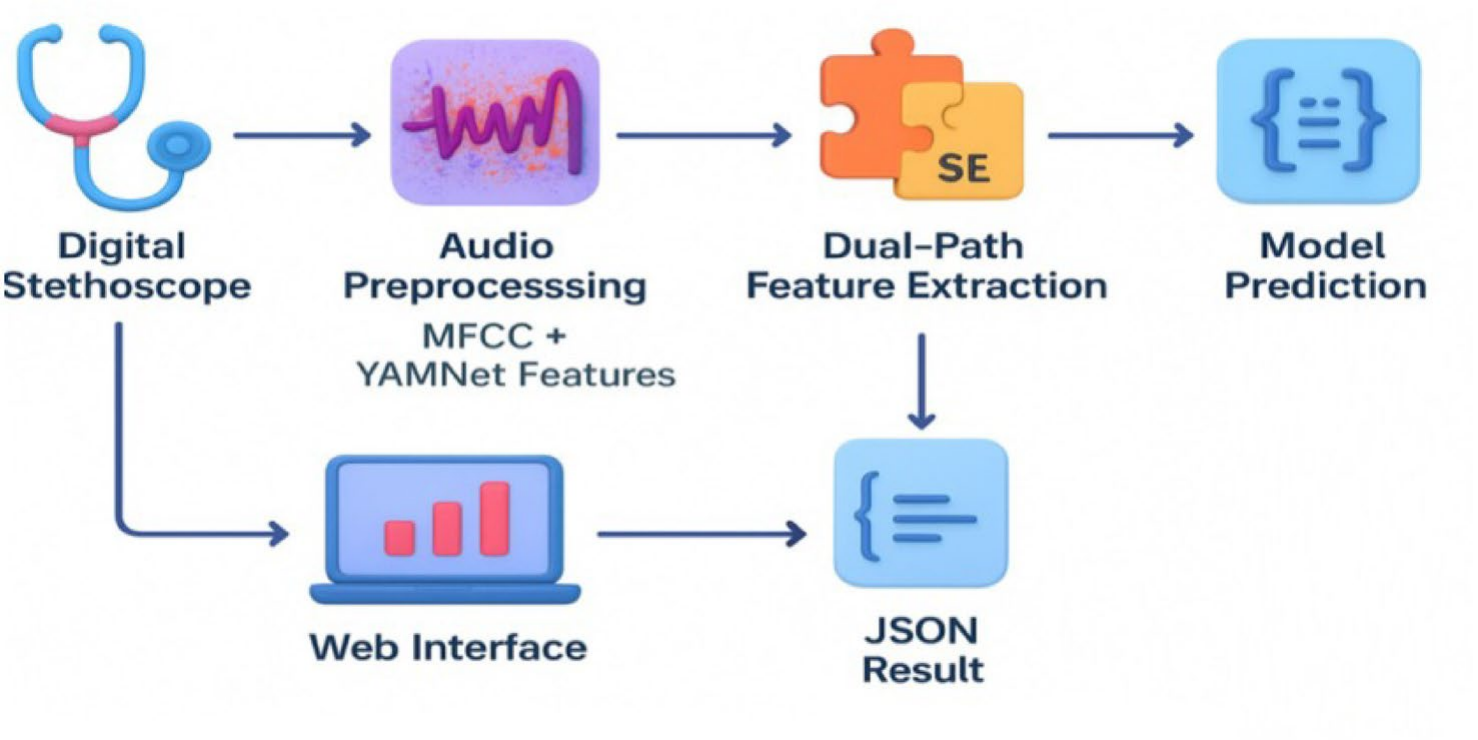
Overview of the proposed asthma detection framework.

The suggested methodology for detecting asthma from respiratory sounds is explained in this section. An overview of the entire process, from data collection to model prediction and deployment, is given in Fig. 1.

As shown in Fig. 1, the pipeline starts with respiratory sound recordings collected via a digital stethoscope. After preprocessing, handcrafted features (MFCCs) and YAMNet embeddings are extracted and fused through a dual-path module with SE blocks. The combined features are classified for asthma detection, and results are delivered through a Flask API for visualization in a web interface, supporting real-world telemedicine use.

### Dataset

A publicly available dataset called “Asthma Detection Dataset (version 2)” curated by Mohammed Tawfik^19^ and made available on the Kaggle platform was used in this study (https://www.kaggle.com/datasets/mohammedtawfikmusaed/asthma-detection-dataset-version-2). Version 2 was last updated approximately one year ago on Kaggle, which aids reproducibility by ensuring clear versioning and traceability.The study in^19^ offers additional insights into respiratory sound analysis and classification using machine and deep learning techniques^31^. To support research on automated respiratory sound analysis and early detection of lung diseases, the Asthma Identification Dataset (version 2) was assembled. These recordings were made using regular mobile phone microphones in non-clinical, real-world contexts, in contrast to numerous clinical datasets that were gathered in controlled hospital settings. Because of this feature, the dataset is extremely relevant for creating scalable and useful diagnostic tools that may be used in telemedicine or community settings. Furthermore, the model’s multiclass structure—which includes asthma, COPD, pneumonia, bronchitis, and healthy cases—offers a broad basis for assessing the model’s resilience to various respiratory disorders. Using mobile phone microphones in real-world settings, 852 audio recordings of voluntary coughs and respiratory noises were made. The files ranged in duration from roughly 1–7 s and were all supplied in .wav format with a constant sample rate of 44.1 kHz. The multiclass-labeled dataset includes both healthy cases and a variety of respiratory diseases. Table 2 provides a summary of how many audio samples are available for each category. The Results section includes representative respiratory sound waveforms and spectrogram examples (Figs. 8 and 9) to further demonstrate the dataset. The temporal and spectral structure of the recordings utilized in this investigation is made clearer by these visuals.Although the dataset exhibits a slight class imbalance (e.g., COPD: 401 samples vs. bronchial: 104 samples), several strategies were adopted during model training to mitigate its impact. First, stratified five-fold cross-validation was employed to preserve the class distribution across folds. In addition, data augmentation techniques were applied to increase variability and improve the representation of minority classes. Furthermore, class weighting was applied during model training so that minority classes receive higher penalties in the loss function. These strategies help reduce potential bias toward majority classes and improve the robustness of the trained model.

**Table 2:** Distribution of respiratory sound samples across different class labels in the dataset.

Class label	Number of samples	p-value
Bronchial	104	0.1
Pneumonia	285	0.01
Asthma	288	0.05
Healthy	133	0.2
COPD	401	0.01

### Pre-processing phase

To prevent any potential data leakage, the dataset was first divided using a stratified five-fold cross-validation strategy. All preprocessing operations were then applied within each fold. Specifically, audio segmentation and data augmentation were performed only on the training portion of the data, while the validation data were processed without augmentation. This strategy ensures that no augmented or segmented samples derived from the same original recording appear in both training and validation sets, thereby maintaining a fair evaluation of the model performance.

#### Data cleaning and standardization

During the preprocessing phase, all respiratory sound recordings in .wav files were loaded and normalized. Each file was resampled to 22,050 Hz, converted to mono, and then trimmed to remove the beginning and ending silence. This sampling rate was selected to balance computational efficiency and signal fidelity, as it preserves the clinically relevant frequency components of respiratory sounds while reducing computational cost, which is commonly adopted in respiratory sound analysis studies^32^. Since clinically significant respiratory sounds usually fall below 8 kHz, downsampling from the original 44.1 kHz preserved all relevant frequency information while reducing training time and file size. This ensured that the model was not affected by extraneous high-frequency noise and instead focused on the acoustic characteristics most indicative of wheezes and crackles. These steps helped ensure uniformity and clarity across the dataset by eliminating redundant data and isolating the most informative respiratory sound segments.

#### Noise reduction

The clarity of respiratory signals was enhanced by reducing ambient and device-related noise using a range of signal processing techniques. After band-pass filtering (100–4000 Hz), amplitude normalization was specifically used to ensure consistent signal intensity across samples. The selected frequency range effectively captures the primary spectral components of respiratory sounds, including wheezes and crackles, while suppressing high-frequency noise and very low-frequency background interference, which is consistent with commonly adopted preprocessing strategies in respiratory sound analysis^32^. These processes preserved crucial sound characteristics like crackles and wheezes while eliminating irrelevant background noise.

#### Audio segmentation and augmentation

After the dataset was divided using stratified five-fold cross-validation, each respiratory recording in the training set was divided into fixed 6-s segments. These segments were long enough to capture complete breathing cycles, which helps enhance model generalization and address the limited dataset size.Segmenting respiratory recordings into fixed-length windows is a commonly adopted strategy in lung sound classification studies to capture complete breathing cycles while maintaining consistent input size for deep learning models^33^.

Techniques for data augmentation were then used to further increase variability and replicate real-world recording conditions. The augmentation techniques were applied only to the training data to avoid potential data leakage. Audio augmentation techniques such as time stretching, pitch shifting, noise injection, and time shifting are widely used in respiratory sound analysis to improve model robustness and reduce overfitting when working with limited datasets^34^. In particular, speed tuning (time stretching) was adjusted between 0.8 $\times$ and 1.2 $\times$ of the initial rate, while pitch scaling was applied at random within ± 2 semitones. Additionally, a factor of 0.005 was used to inject low-level Gaussian noise. Additionally, time shifting was accomplished by varying the waveform’s position inside the segment at random. In order to maintain the perceived quality of respiratory sounds while adding enough variability to lessen overfitting and increase the model’s robustness, these parameter ranges were empirically chosen through early testing.

### Feature extraction

To distinguish asthma from non-asthma instances, a dual-path feature extraction approach was used, combining numerical auditory descriptors and spectrogram-based representations. All audio files were converted to mono and resampled before analysis to ensure uniformity. Librosa was used with a 40-band Mel filter bank to extract Mel-frequency cepstral coefficients (MFCCs), zero-crossing rate (ZCR), chromagrams, RMS energy, spectral centroids, rolloff, bandwidth, and log power spectrograms. By coarsening at higher frequencies to mirror human hearing perception, this filter architecture maintains computational efficiency while providing greater resolution at low frequencies, when important respiratory events like wheezing are noticed it^32^.

Important spectral and temporal patterns that help identify problematic respiratory sounds linked to asthma, like persistent wheezes or brief crackles, are encoded in the retrieved features. Every feature type has intrinsic limits, such as the spectral centroid’s sensitivity to noise, ZCR’s susceptibility to non-respiratory aberrations, and MFCCs’ potential inability to discern fast temporal fluctuations^33^. However, these characteristics are very complementary; using a variety of descriptors guarantees a more reliable and complex description of respiratory audio, in line with proven methods in earlier clinical and machine learning investigations^35,36^. Numerous studies show that MFCC and ZCR are quite good at detecting wheezing, and their combination with Gaussian Mixture Models results in excellent sensitivity for noises associated with asthma. Thus, the feature set and analytical methodology of the current investigation align with recognized best practices in pulmonary sound analysis^31^. Feature importance analysis was later performed using SHAP values to identify the most informative handcrafted acoustic descriptors and mitigate redundancy among extracted features.

#### Chromagram

The intensity of the signal’s 12 different pitch classes is captured by this characteristic. It helps distinguish between tone components and reflects harmonic content. For every audio sample, the chroma vectors’ standard deviation was calculated.

#### Root mean square energy (RMS)

The signal strength over time is represented by RMS energy, which provides a reliable indicator of amplitude variations and energy peaks that are particularly pertinent to breath analysis.

#### Spectral centroid

This correlates with the perceived brightness of the sound and shows the “center of mass” of the spectrum. It aids in the description of wheezing’s high-frequency content.

#### Spectral roll-off

The frequency at which 85% of the total spectral energy is contained is known as the roll-off. It helps detect irregular breathing and records the change in spectral energy.

#### Spectral bandwidth

Calculates the spectrum’s dispersion around the centroid. Noisier or more erratic respiratory events, like crackles or wheezes, are frequently associated with a wider bandwidth.

#### Zero-crossing rate (ZCR)

The frequency with which the signal changes sign (crosses the zero amplitude line) is measured by the zero-crossing rate. In addition to helping identify abrupt changes that could happen during aberrant respiratory events like crackles, it is a helpful indicator of a signal’s noisiness or roughness.

#### Mel-frequency cepstral coefficients (MFCCs)

Mel-Frequency Cepstral Coefficients (MFCCs) are widely used in speech and respiratory sound analysis due to their ability to compactly represent the power spectrum on a perceptual scale. In this study, a six-stage processing pipeline was implemented to extract 20 MFCCs from each audio signal. The process begins with pre-emphasis, where a high-pass filter enhances high-frequency components and compensates for signal attenuation, thereby improving the signal-to-noise ratio. The preprocessed waveform is then divided into short overlapping frames, typically 25 ms in length with a 10 ms stride, to capture the quasi-stationary nature of lung sounds. Each frame is smoothed using a Hamming window to reduce spectral leakage and minimize discontinuities at the frame boundaries. The Fast Fourier Transform (FFT) converts the time-domain frames into their frequency-domain representation, emphasizing the spectral content of respiratory events such as wheezes and crackles. The resulting spectrum is passed through a set of 40 triangular bandpass filters spaced along the Mel scale to approximate the nonlinear frequency response of the human auditory system. Finally, a Discrete Cosine Transform (DCT) is applied to the logarithmic filter bank energies to decorrelate the features, and the first 20 coefficients are retained to form the compact MFCC representation used for subsequent classification.

#### Log power spectrogram analysis

The Short-Time Fourier Transform (STFT) was used to create log-scaled power spectrograms, which were then logarithmically scaled using librosa. Power_to_db in order to improve the interoperability of respiratory sound characteristics in the frequency domain. These spectrograms make it possible to identify acoustic biomarkers like crackles and wheezes by clearly visualizing energy concentration with time and frequency.

#### Dataset splitting and validation strategy

**Fig. 2:**
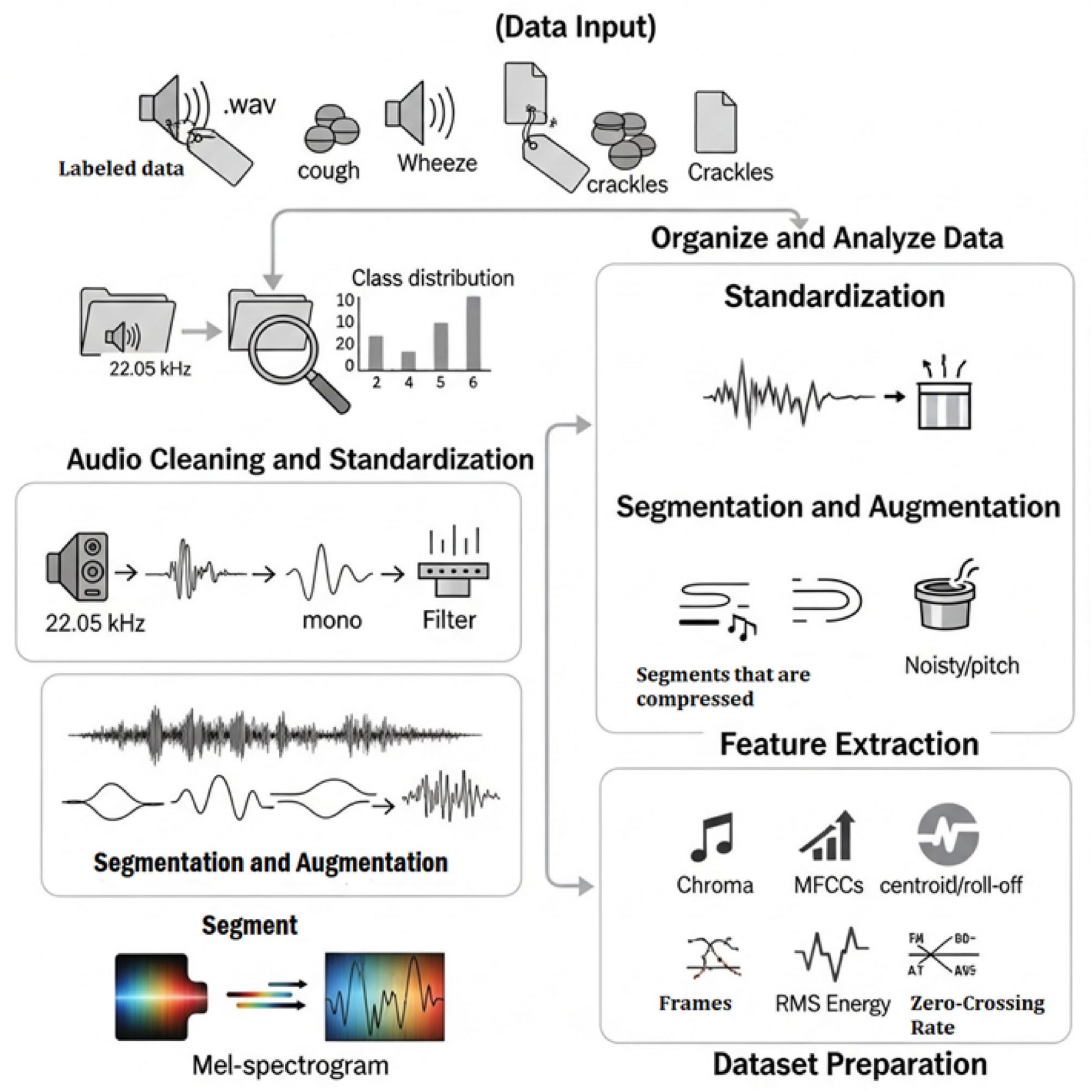
Comprehensive preprocessing framework for labeled respiratory sound data, including standardization, filtering, and overlapping segmentation.

To ensure reliable evaluation and prevent overfitting, the dataset was split using Stratified K-Fold Cross-Validation with five folds. This method preserves class balance across splits, which is essential for multiclass classification. In each fold, the model was trained on 80% of the data and validated on the remaining 20%. Final performance metrics were averaged over the five folds.To avoid potential data leakage, dataset splitting was performed prior to segmentation and augmentation. All preprocessing operations were conducted separately within each fold of the cross-validation process (Fig. 2).

**Algorithm 1:**
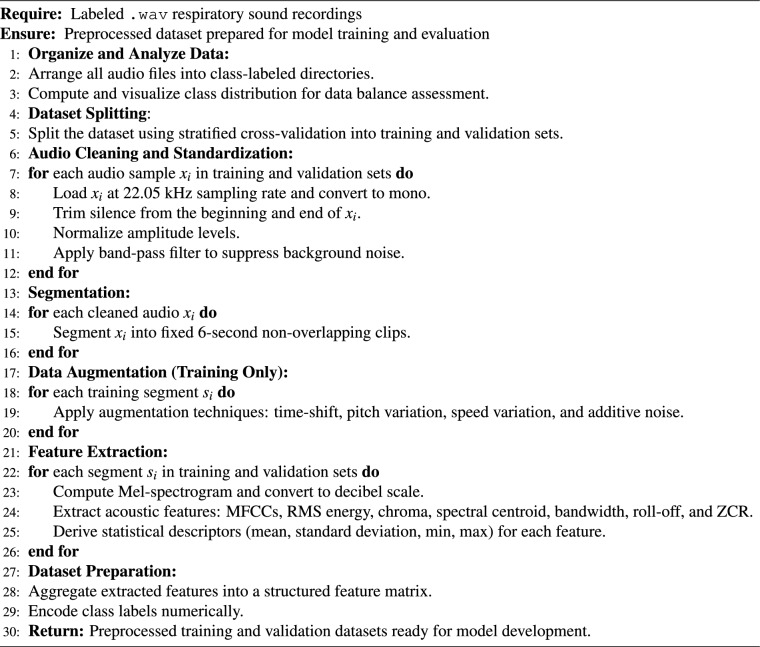
Preprocessing of respiratory sound dataset for asthma detection.

## Methodology

**Fig. 3:**
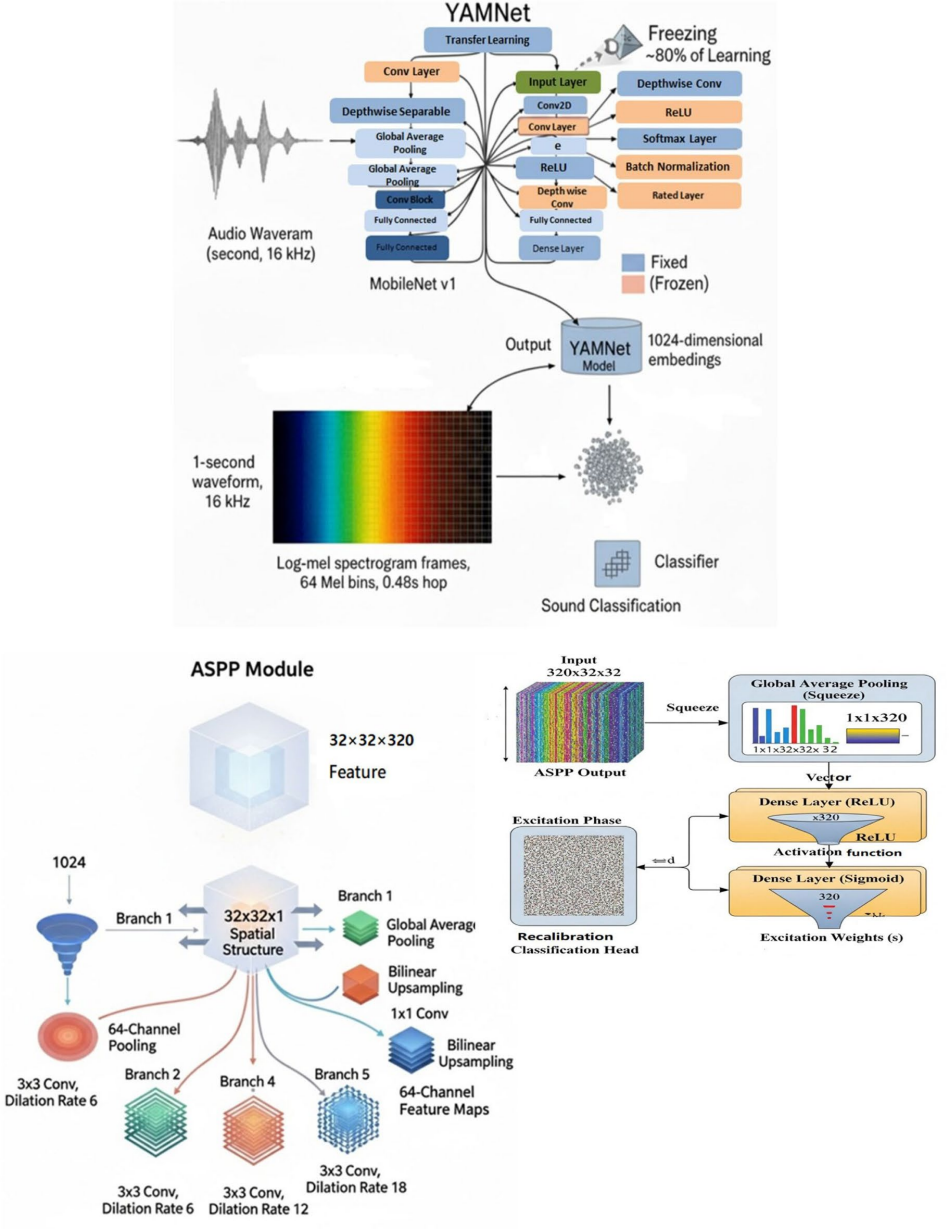
The dual-path respiratory sound categorization framework’s overall architecture. The pipeline incorporates a multi-layer perceptron for final illness classification, an ASPP module for multi-scale feature extraction, a Squeeze-and-Excitation block for adaptive channel recalibration, and YAMNet transfer learning for deep audio embeddings.

In this study, both learnt and hand-crafted representations from respiratory audio signals were captured using a dual-path feature extraction technique. Pretrained deep embeddings capture more intricate temporal–spectral patterns, while hand-crafted features (such as MFCC, Chromagram, and ZCR) offer interpretable auditory indications of respiratory pathology. This method was chosen to combine the advantages of the two feature types. Prior research has demonstrated that these hybrid approaches perform better in respiratory sound classification^37^ than either feature type alone. They also exhibit superior generalization and increased resistance to noise across a variety of recording settings^34,38^. Respiratory sounds refer to the raw breathing and coughing recordings, while acoustic features denote the processed descriptors extracted from these signals (e.g., MFCCs, chromagram, ZCR). Throughout the paper, respiratory sounds describe the input signals, and acoustic features refer to their derived representations for classification. Each audio recording was first denoised, then divided into fixed 6-s segments and augmented in order to improve model generalization even more. After that, two pipelines were used in parallel for processing:

- In the first step, the Librosa package was used to extract a set of manually created spectral and temporal properties. These included root mean square energy (RMS), bandwidth, MFCCs, chromagrams, log-scaled power spectrograms, zero-crossing rate (ZCR), spectral centroid, and spectral rolloff. Model comparison and exploratory analysis were the main uses for these attributes.
- The second method made use of YAMNet, a pretrained audio classification model developed by Google and trained on the extensive AudioSet ontology, in conjunction with deep semantic embedding. Every 1-s mono waveform was transformed into a sequence of 1024-dimensional embeddings after being resampled to 16 kHz. YAMNet was selected because of its effective MobileNetV1 backbone and wide acoustic coverage, which enable it to generalize well to subtle biological signals. It obtains fine-grained temporal–spectral patterns by working with log-mel spectrogram inputs, which are essential for identifying aberrant respiratory sounds like asthmatic crackles and wheezes. The suggested deep learning architecture, which comprised an Atrous Spatial Pyramid Pooling (ASPP) module for multi-scale feature extraction, a Multi-Layer Perceptron (MLP) classifier, and a Squeeze-and-Excitation (SE) block for channel-wise attention tuning, was then fed these embeddings. Figure 3 shows the general architecture of the suggested dual-path deep learning system. For the final asthma prediction, it combines hand-crafted audio descriptors with pretrained YAMNet embeddings, attention-enhancing SE blocks, and a Multi-Layer Perceptron classifier.

### Input and preprocessing

To maintain dataset homogeneity while maintaining the essential respiratory frequency range (100–4000 Hz) without incurring needless computational costs, all audio signals were converted to mono and resampled to 16 kHz. Every clip was truncated or zero-padded to 1 second (16,000 samples). The audiomentations software was then used to apply data augmentation, which included Gaussian noise insertion, pitch shifting (± 2 semitones), and temporal stretching (0.9–1.1 $\times$).

### YAMNet embedding extraction: base model (YAMNet transfer learning)

YAMNet, a pretrained deep audio classification model developed by Google and trained on the AudioSet ontology, was used to extract high-level semantic characteristics from respiratory sounds. Rich time-frequency representations in a variety of auditory domains can be recorded by the MobileNet v1 architecture, which serves as the foundation for YAMNet.

The MobileNetV1 backbone employed in YAMNet relies on depthwise separable convolutions, which decompose standard convolution operations into depthwise and pointwise convolutions. This architectural design significantly reduces computational complexity and the number of trainable parameters while maintaining strong feature extraction capability. Each convolutional block is followed by batch normalization and ReLU activation functions, enabling the network to progressively learn hierarchical audio representations ranging from low-level spectral patterns to higher-level semantic acoustic features. The model processes a 1-s mono waveform that has been resampled to 16 kHz using a sequence of depthwise separable convolutional layers, global average pooling, and fully connected projections. YAMNet internally transforms the input waveform into log-mel spectrogram frames, each with a windowing hop of 0.48 s and 64 Mel bins.The acoustic content of a single frame in the latent space is represented by each of the 1024-dimensional embeddings created by passing these frames through the network. We determine the average embedding across all frames in order to produce a fixed-length representation for further classification:

1 $$\begin{aligned} e = \frac{1}{T} \sum _{t=1}^{T} e_t, \qquad e_t \in \mathbb {R}^{1024} \end{aligned}$$

where $e \in \mathbb {R}^{1024}$, is the final embedding used as input to the downstream classifier. To improve transfer learning’s effectiveness in our particular model:

- YAMNet’s topmost classification layer was eliminated when it was initialized with include top = False. This enables it to be utilized just as a potent respiratory sound feature extractor.
- Eighty percent of the layers in the underlying YAMNet model were frozen while it was being trained. While concentrating the training on the recently added layers of our model to adjust to the particular respiratory disease classification job, this guarantees that the vast amount of pre-learned acoustic knowledge within YAMNet is maintained. This percentage was empirically established following initial tests, which showed that freezing more than 90% of the layers hindered the model’s capacity to adjust to domain-specific respiratory sound patterns, while freezing fewer layers (e.g., 50–60%) resulted in overfitting because of the comparatively short dataset size. As a result, freezing 80% of the layers offered a fair compromise, allowing the remaining layers to focus on respiratory signals associated with asthma while maintaining YAMNet’s broad acoustic information. Compared to alternative freezing ratios, this approach enhanced validation performance, stabilized training, and decreased computing cost.By using YAMNet’s transfer learning capacity and reducing the need for large labeled respiratory datasets, this method allows our model to take advantage of pretrained auditory knowledge. Both temporal and spectral hints are preserved in the retrieved embeddings, which are essential for identifying anomalous patterns like crackles and wheezing.

#### Feature fusion strategy

To integrate the complementary information captured by deep embeddings and handcrafted acoustic descriptors, a feature-level fusion strategy is adopted.

Let $z_y \in \mathbb {R}^{1024}$ denote the embedding vector extracted from the pretrained YAMNet model for each audio segment. In parallel, handcrafted acoustic features $z_{handcrafted}$ are computed using Librosa, including MFCCs, chromagram, zero-crossing rate (ZCR), spectral centroid, spectral bandwidth, spectral contrast, and spectral roll-off.

The two feature representations are fused using feature concatenation:

$$Z = [z_y , z_{handcrafted}]$$

where *Z* represents the combined feature vector that integrates both deep semantic information and interpretable acoustic descriptors. This fused representation is then forwarded to the subsequent feature enhancement stage, where the Atrous Spatial Pyramid Pooling (ASPP) module captures multi-scale acoustic patterns. The resulting features are further refined using a Squeeze-and-Excitation (SE) block to adaptively recalibrate channel-wise responses before being passed to the Multi-Layer Perceptron (MLP) classifier for final prediction.

### Reshaping and ASPP module

**Fig. 4:**
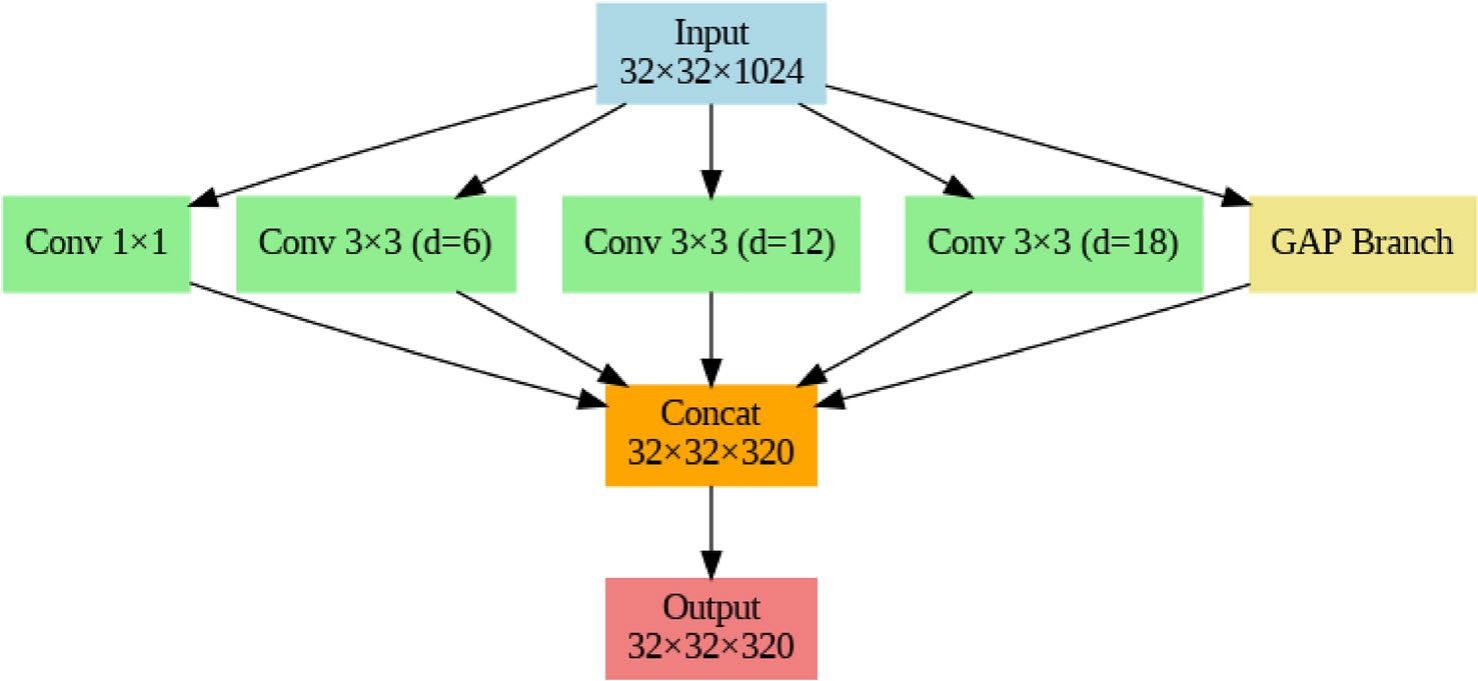
Atrous spatial pyramid pooling (ASPP) module.

The 1024-dimensional feature vector of each 1-s audio clip was taken out of YAMNet and converted into a 2D spatial structure that was 32 $\times$ 32 $\times$ 1. This reshaping allows convolutional layers to better capture local and contextual patterns while maintaining the intrinsic embedding values by mimicking an image-like representation. YAMNet embeddings are developed from log-mel spectrograms, which naturally encode these associations, even though the strict temporal or spectral order may not be explicitly maintained in this format. The model’s capacity to represent multi-scale dependencies is further improved by the inclusion of an Atrous Spatial Pyramid Pooling (ASPP) module, which also helps to mitigate any slight ordering ambiguities that may have been generated during reshaping. To extract characteristics at various scales, the ASPP block uses dilated convolutions, which enlarge the receptive field without raising parameters. The ASPP module of our architecture consists of five parallel branches:

- A convolution measuring 1 $\times$ 1 and having a dilation rate of 1.
- Three 3 $\times$ 3 convolutions with corresponding dilation rates of 6, 12, and 18.
- To match the spatial dimensions, a 1 $\times$ 1 convolution and bilinear upsampling are performed after a global average pooling layer. Every convolutional branch generates a 64-channel feature map. A ReLU activation function is applied to each convolutional layer in these branches, and “same” padding is employed to preserve spatial dimensions. Every one of these convolutional branches produces a feature map with 64 channels. A 32 $\times$ 32 $\times$ 320 output tensor is created by concatenating all outputs along the channel dimension, as shown in Fig. 4.

2 $$\begin{aligned} \mathbb {R}^{32 \times 32 \times 320} \ni \textrm{Concat}(y_1, y_2, y_3, y_4, y_5), \quad X = \textrm{ASPP}(X) \end{aligned}$$

The model can identify intricate and varied acoustic patterns like wheezing, crackles, and breathing disturbances that may occur at various temporal or spectral scales thanks to this ASPP-enhanced representation, which blends local features with global context. Dilation rates 1, 6, 12, and 18 were chosen because they offer a range of receptive fields that are well-balanced, ranging from fine-grained local features to more general contextual information. DeepLabv3 models have made extensive use of this particular combination and validated it^9^. Recent studies on atrous rate optimization have shown how crucial it is to adjust dilation rates in accordance with the properties of the input data to attain the best possible effective receptive fields arXiv. In contrast to dilation ranges that were too little or too large, this set of rates produced better stability during training and higher validation accuracy in our studies. As a result, these settings capture global respiratory patterns over time and frequency while maintaining important auditory characteristics like wheezes and crackles.

### Squeeze-and-excitation (SE) block

In order to adjust channel-wise feature responses and enhance the model’s representational strength, we included a Squeeze-and-Excitation (SE) block after the ASPP module, which records multi-scale contextual information. There are three phases to the SE block’s operation:

- Squeeze: To create a compact descriptor vector of shape (1 $\times$ 1 $\times$ 320), the spatial dimensions (32 $\times$ 32) of each of the 320 feature maps are globally averaged. This creates a summary of the global distribution of every channel.
- Excitation: This description passes through two completely linked layers to provide a set of modulation weights, one for each channel. The first is a Dense layer that applies a ReLU activation and decreases the dimensionality of the channels by a predetermined reduction ratio . The second is another Dense layer that applies a sigmoid activation and expands the dimensionality back to the initial number of channels (320). These weights take into account inter-channel relationships and determine each feature map’s relative value.
- Recalibration: Using the learnt channel weights, the original feature maps are rescaled (element-wise multiplication) so that the network can reduce noise or unnecessary patterns and highlight discriminative features like crackle bursts or wheezing harmonics.

**Fig. 5:**
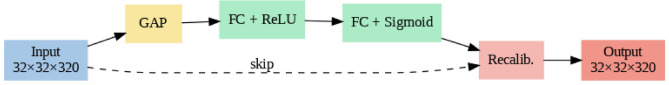
Squeeze-and-excitation (SE) block for channel-wise feature recalibration.

Mathematically, let $\textbf{X} \in \mathbb {R}^{320 \times 32 \times 32}$ be the ASPP output. The SE block produces a reweighted output $\tilde{\textbf{x}} \in \mathbb {R}^{320 \times 32 \times 32}$:

$$\textbf{X} \cdot \textbf{s} = \tilde{\textbf{X}} \quad \text {where} \quad \textbf{s} = \sigma \left( W \cdot \textbf{X}_c(i,j) \right)$$

where $\textbf{z} \in \mathbb {R}^{320}$ is the squeezed descriptor, $\delta$ and $\sigma$ are ReLU and sigmoid activations, respectively, and $\textbf{s} \in \mathbb {R}^{320}$ is the excitation vector. Each feature map $\textbf{X}_c$ is scaled by its corresponding excitation weight $\textbf{s}$, resulting in an enhanced feature representation. With optimum channel-wise activations and the same spatial structure $(32 \times 32 \times 320)$, this SE-enhanced tensor aids the model in focusing on the most instructive spectral–temporal patterns for asthma classifications as shown in the Fig. 5. In pulmonary acoustics, this mechanism adaptively enhances channels that record the narrow-band tone components of wheezes and channels sensitive to high-frequency, short-duration bursts that correspond to crackles. It also mutes channels that are dominated by irrelevant or background noise. In addition to decreasing false activations, this selective recalibration enhances the model’s capacity to identify clinically significant events, which raises classification accuracy overall.

### Classification head

**Fig. 6:**
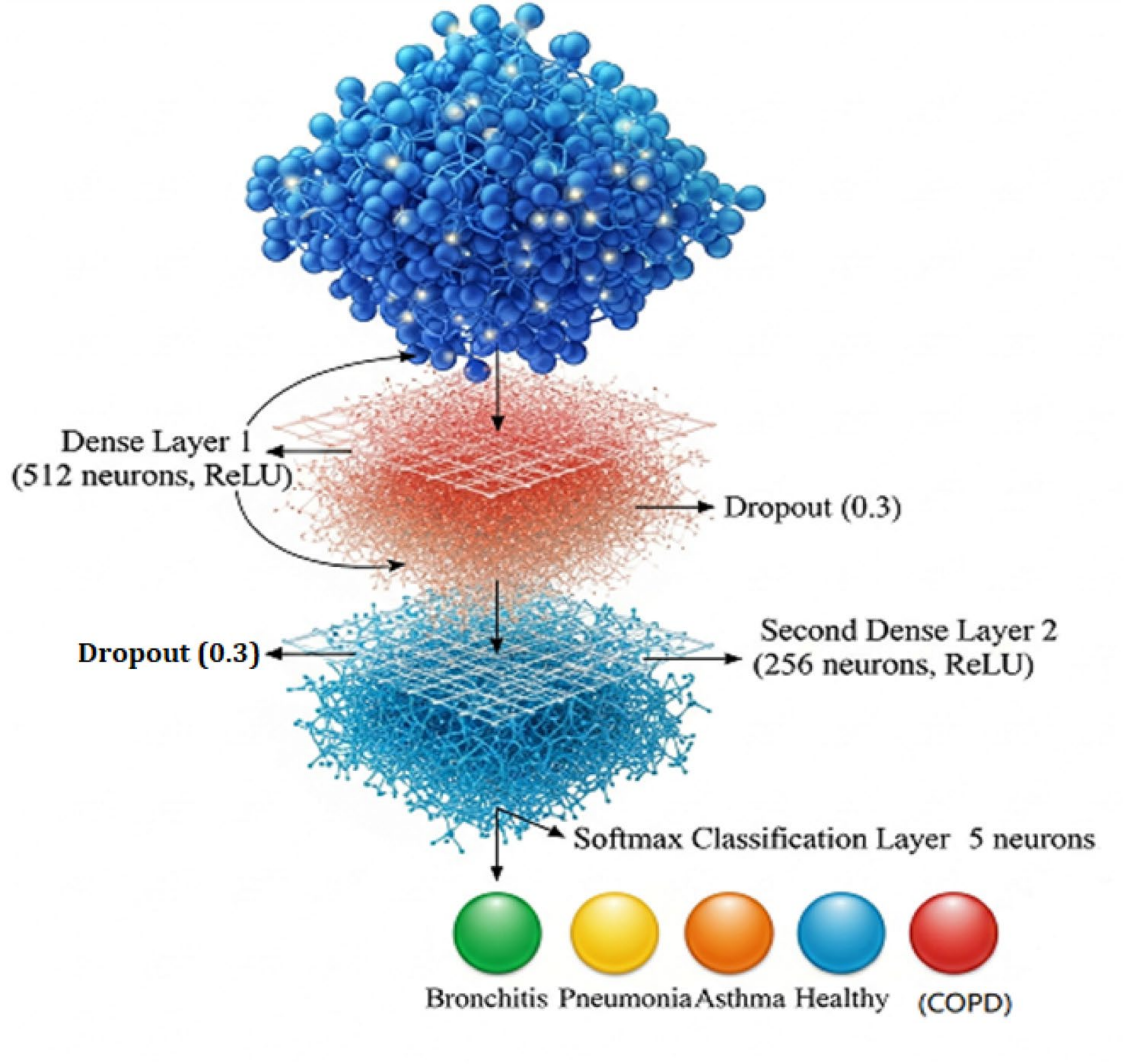
The proposed deep learning architecture using dense layers, dropout, and a final softmax layer for five-class respiratory disease classification.

A one-dimensional vector is created by flattening the resulting tensor, which has dimensions of 32 $\times$ 32 $\times$ 320, after the ASPP module has improved the spatial properties. Through this transformation, the high-dimensional spatial representation is transformed into a format that fully connected layers can process. A Multi-Layer Perceptron (MLP), which is intended to describe intricate, non-linear interactions among the retrieved acoustic data, is then given the flattened vector. The MLP consists of two dense layers that follow one another:

- A dropout layer with a dropout rate of 0.3, which acts as a regularization strategy to lessen overfitting by randomly deactivating a portion of neurons during training, comes after the first dense layer, which has 512 neurons and a ReLU activation function to add non-linearity.
- 256 neurons make up the second dense layer, which is followed by ReLU activation and dropout (0.3). This layer maintains the model’s generalizability while further honing the learned feature representations.
- Early empirical testing established the sizes of the fully linked layers (512 and 256 neurons), finding that lower configurations ($\le$ 256) resulted in underfitting while bigger ones (1024–512) increased computing cost without obvious performance advantages. Because of the balanced trade-off offered by the 512–256 structure, the network was able to preserve generalization while capturing intricate non-linear interactions in the embeddings. After initial tests revealed that higher rates ($\ge 0.5$) resulted in unstable convergence and lower rates ($\le 0.2$) did not effectively avoid overfitting, dropout with a rate of 0.3 was chosen as a regularization strategy. This setup is also in line with earlier deep learning research on audio-based illness diagnosis, which found that stable training and increased robustness were achieved with modest layer sizes and dropout as shown in Fig. 6.

The output of the MLP is passed through a softmax classification layer consisting of 5 neurons, each representing one of the target respiratory conditions:

- Bronchitis
- Pneumonia
- Asthma
- Healthy
- Chronic Obstructive Pulmonary Disease (COPD)

The softmax function converts the output logits into a probability distribution across the five classes. The class associated with the highest probability is selected as the final prediction. This classification head enables the model to effectively translate rich acoustic representations into clinically relevant diagnoses, bridging the gap between feature extraction and decision-making in a robust and interpretable manner.

### Performance evaluation metrics

A wide range of assessment metrics and visualization tools used to thoroughly assess the performance of the suggested deep learning model for multiclass respiratory sound categorization. These comprised confusion matrices, feature attribution analysis using SHAP, accuracy, precision, recall, F1-score, and area under the receiver operating characteristic curve (AUC). The sklearn.metrics module was used to calculate all metrics. The classification performance of the proposed model was evaluated using a set of standard metrics derived from the confusion matrix: Accuracy, Precision, Recall (Sensitivity), and F1-Score. These metrics quantify various aspects of the model’s predictive capability, particularly in multiclass and imbalanced data settings.

- Accuracy quantifies the overall proportion of correct predictions, including both true positives and true negatives, relative to the total number of predictions:
  3 $$\begin{aligned} Accuracy = \frac{TP + TN}{TP + TN + FP + FN} \end{aligned}$$
- Precision reflects the proportion of correctly predicted positive samples among all samples classified as positive:
  4 $$\begin{aligned} Precision = \frac{TP}{TP + FP } \end{aligned}$$
- Recall (Sensitivity) represents the proportion of actual positive samples that were correctly classified:
  5 $$\begin{aligned} Recall = \frac{TP}{TP + FN } \end{aligned}$$
- F1-Score provides a balanced measure between precision and recall, and is particularly informative when the dataset exhibits class imbalance:
  6 $$\begin{aligned} F_1 = 2 \times \frac{Precision \times Recall}{Precision + Recall} \end{aligned}$$
- Using a One-vs-Rest (OvR) classification technique, the Area Under the Receiver Operating Characteristic Curve (AUC-ROC) was used to assess the model’s capacity to differentiate between the five respiratory classes: bronchial, pneumonia, asthma, healthy, and COPD. This method compares each class’s separability to all others. The True Positive Rate (TPR) and False Positive Rate (FPR) for every class were calculated as follows:
  7 $$\begin{aligned} FPR = \frac{FP}{FP + TN },\ TPR = \frac{TP}{TP + FN } \end{aligned}$$

Using the sklearn.metrics package, all performance measures were obtained straight from the confusion matrix. In order to retain class distribution across folds and solve the dataset’s inherent class imbalance (for example, COPD had 401 samples compared to Healthy, which had 133), we used stratified five-fold cross-validation. Furthermore, macro-averaged precision, recall, F1, and AUC ratings were provided. By giving each class the same weight regardless of how frequently it occurs, this averaging technique offers a more equitable evaluation of model performance in unbalanced multiclass environments. Although weighted accuracy was not specifically used, stratified splitting and the macro-averaged results successfully reduce the consequences of imbalance and guarantee a reliable assessment for all respiratory illnesses.

**Fig. 7:**
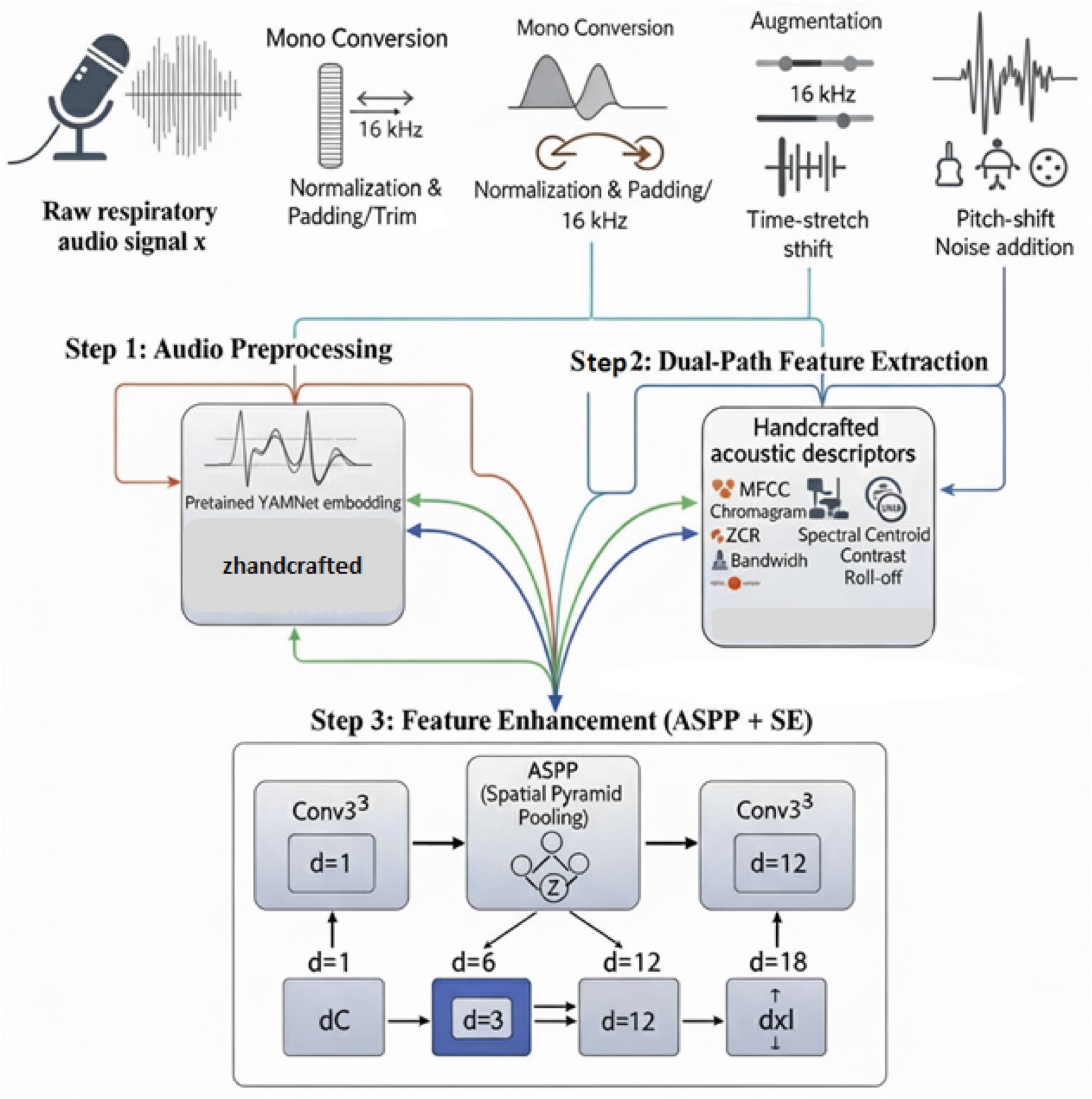
Dual-path feature extraction and enhancement for audio classification.

The model’s internal decision-making process was interpreted using SHAP (SHapley Additive Explanations). Each input feature, in this example the 1024-dimensional YAMNet embedding, is given a contribution score based on the final prediction. employing shape. A subset of the validation data was used to calculate the KernelExplainer and SHAP values. In order to differentiate between respiratory disorders like asthma and normal breathing, a summary plot showed which latent factors had the most influence on predictions. This step on interpretability improves transparency by providing information about how the model makes decisions that are clinically relevant. Algorithm 2 was restructured into modular parts (preprocessing, feature extraction, ASPP, SE, and classification) to improve readability. The end-to-end pipeline is also graphically summarized in a schematic flowchart (Fig. 7).

**Algorithm 2:**
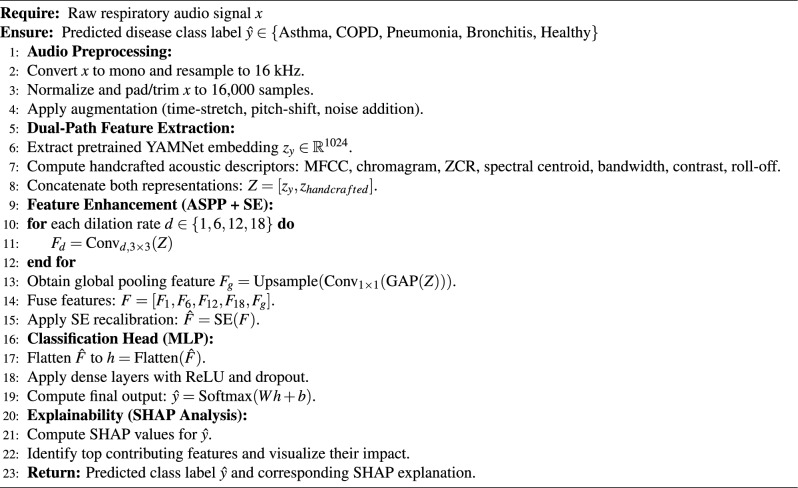
Dual-path respiratory sound classification using YAMNet, ASPP, SE, and SHAP.

## Results

### Experimental setup

TensorFlow/Keras was used to implement all experiments in Python within a Jupyter Notebook environment. A hybrid computational setup was used: large-scale training and evaluation were done on cloud-based platforms (Google Colab and Kaggle) with access to NVIDIA Tesla T4 GPUs (16 GB VRAM), while initial preprocessing and model prototyping were done on a local workstation with an 11th Gen Intel®Core™i7-11800H CPU @ 2.30 GHz and 16 GB RAM. This configuration guaranteed consistency, decreased computing cost, and expedited training.

All audio signals were transformed into 1-s mono waveforms sampled at 16 kHz before model fitting, in compliance with the required input format of YAMNet. A real-time data generator was integrated with a unique pre-processing pipeline that featured zero-padding, amplitude normalization, and silence cutting for effective training of each audio clip. The audiomentations library was used to dynamically apply online data augmentation during training in order to enhance generalization and replicate real-world variability. This process involved Gaussian noise injection, pitch shifting (± 2 semitones), and mild time-stretching (± 10%) to make sure that adjustments to the recording parameters wouldn’t affect the underlying breathing patterns. The ranges that were chosen were determined by combining empirical pilot testing on our dataset with previous studies in respiratory and voice sound analysis. While maintaining the clinical integrity of important acoustic cues, like wheezes and crackles, which are essential for diagnosing asthma, these values added enough variety to enhance generalization. The categorical cross-entropy loss function was employed due to the multi-class nature of the challenge, and the Adam optimizer was utilized to train the model with a learning rate of 0.0001. 32-sample mini-batches were used for training over a maximum of 50 epochs. Additional callbacks were used for training monitoring and model checkpointing, and early stopping (patience = 5) was implemented based on validation loss to avoid overfitting.

StratifiedKFold was used to perform a stratified five-fold cross-validation for assessment, guaranteeing a balanced distribution of classes across the training and validation sets. For the final evaluation, metrics such as accuracy, precision, recall, F1-score, and AUC were calculated per fold and averaged. SHAP was used post-training on YAMNet embeddings to enhance model interpretability by enabling feature attribution and providing insight into the most significant latent dimensions. All things considered, the configuration guarantees a strong, interpretable, and repeatable framework that can manage data imbalance and unpredictability in actual respiratory sounds.

The computational complexity of the proposed model is mainly dominated by the pretrained YAMNet feature extractor, which is based on the lightweight MobileNetV1 architecture designed for efficient audio processing. The additional ASPP and SE modules introduce only a small number of parameters compared to the backbone network.

In our experimental setup, the model was trained on an NVIDIA Tesla T4 GPU. The average training time was approximately 10 min per fold during the five-fold cross-validation process. The inference time for a single audio sample is less than 1 s, demonstrating that the proposed framework can efficiently operate in real-time respiratory sound analysis and telemedicine applications.

The proposed model contains approximately 5 million parameters, including about 1.2 million trainable parameters and 3.8 million non-trainable parameters. The non-trainable parameters mainly correspond to the frozen layers of the pretrained YAMNet feature extractor, while the trainable parameters belong to the additional ASPP, SE, and classification layers introduced in the proposed architecture. This configuration enables the model to leverage pretrained audio representations while maintaining a relatively lightweight number of trainable parameters.

### Qualitative results

**Fig. 8:**
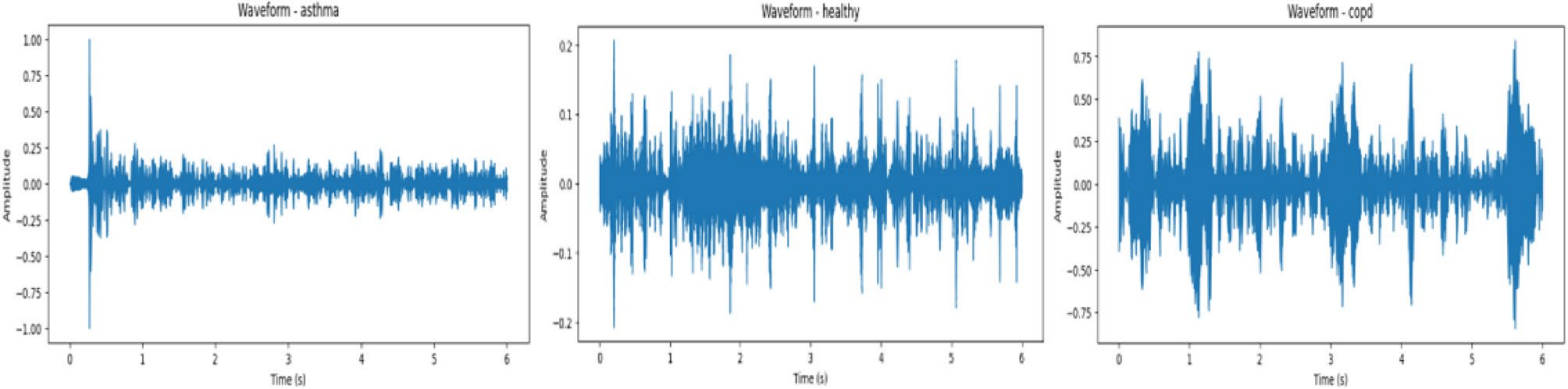
Waveform plots for Healthy, asthma and copd audio recordings after trimming and standardization.

**Fig. 9:**
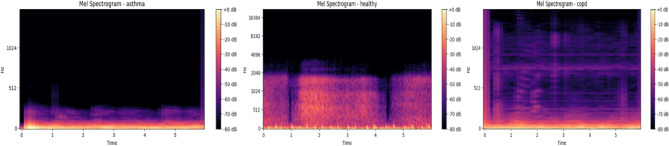
Mel spectrogram representation for respiratory audio signal, highlighting frequency-based structure after augmentation.

A number of visualization techniques were used to enhance comprehension of the suggested model’s discriminative ability. These statistics, which were also included in the preprocessing phase, demonstrate how important acoustic information aids in classification and attest to its preservation. Figure 8 illustrates how the temporal structure of waveforms varies significantly between conditions: COPD samples show lower amplitudes and flatter, longer breathing cycles, which reflect airflow obstruction; asthma signals have irregular peaks and abrupt fluctuations that correspond to wheezing and unstable airflow; and healthy recordings are smoother and more periodic. The mel-spectrogram of an asthma case is shown in Fig. 9, where wheezes are identified by persistent high-frequency energy bands. Conversely, COPD patients exhibit more limited and less varied spectral distributions, while healthy individuals exhibit steady low-frequency periodic patterns. The model’s ability to accurately distinguish asthma from acoustically comparable illnesses is explained by these qualitative results, which also concur with accepted clinical findings. By showing that the model is learning clinically significant representations rather than depending on erroneous correlations, they offer visual evidence to support the quantitative measures.

**Fig. 10:**
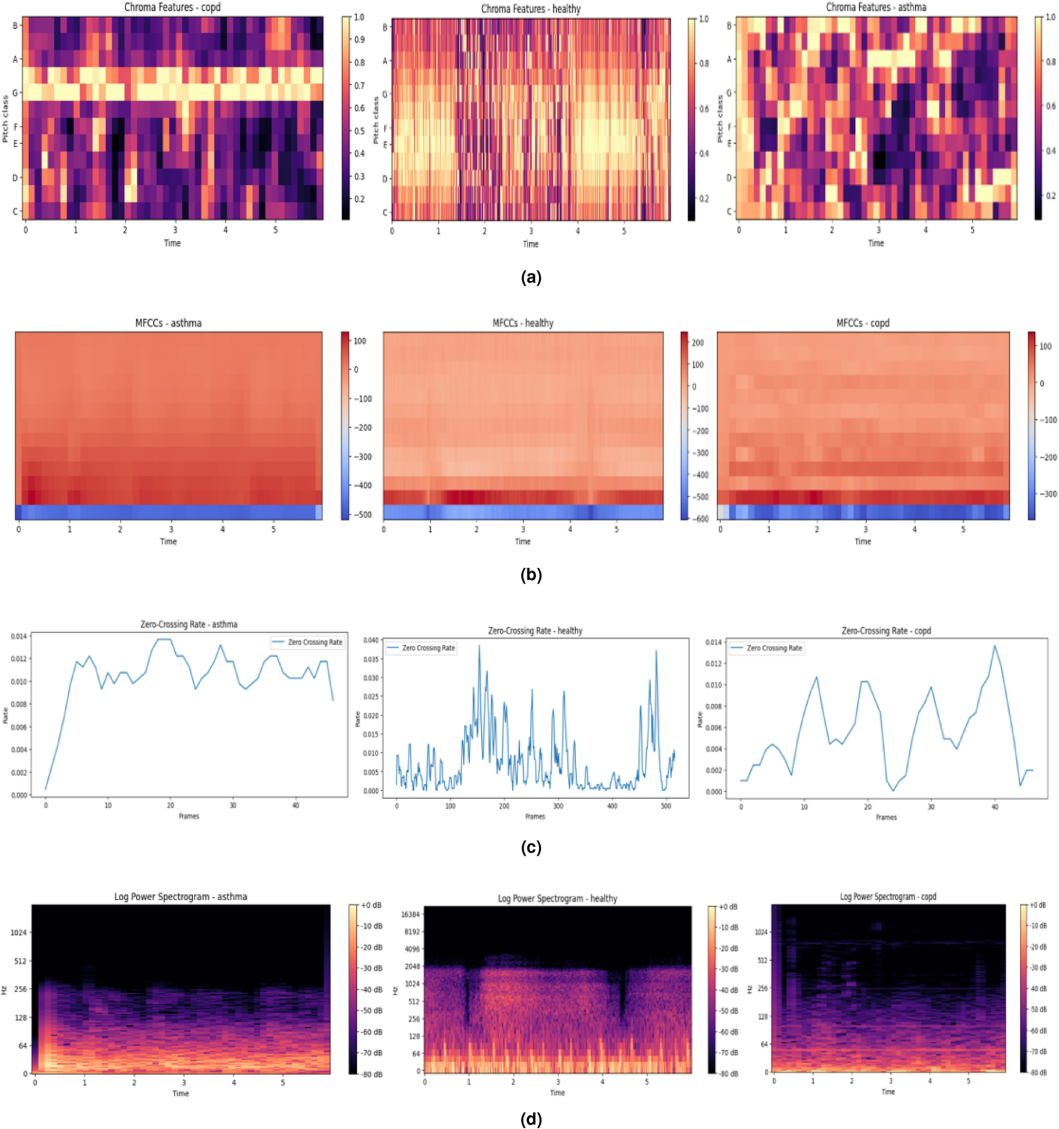
Visual representation of extracted features from respiratory signals: (**a**) Chromagram, (**b**) MFCC, (**c**) ZCR, (**d**) Log Power Spectrogram Analysis.

The extracted feature maps are displayed in Fig. 10a–d to help visualize the role that particular features play in classification.Tonal distribution is revealed by the chromagram (Fig. 10a), which can aid in the differentiation of vocal portions. The MFCC (Fig. 10b) records spectral structure and changes over time, while the Zero Crossing Rate(Fig. 10c) emphasizes sudden signal transitions. Energy concentration is seen via the Log Power Spectrogram (Fig. 10d), which is helpful for spotting signal bursts or noise-like structures. These qualitative assessments confirm that the information required to differentiate between respiratory diseases is preserved and improved via the preprocessing pipeline and feature extraction stages. They also show how the model uses both frequency-domain and time-domain patterns to produce precise predictions.

**Fig. 11:**
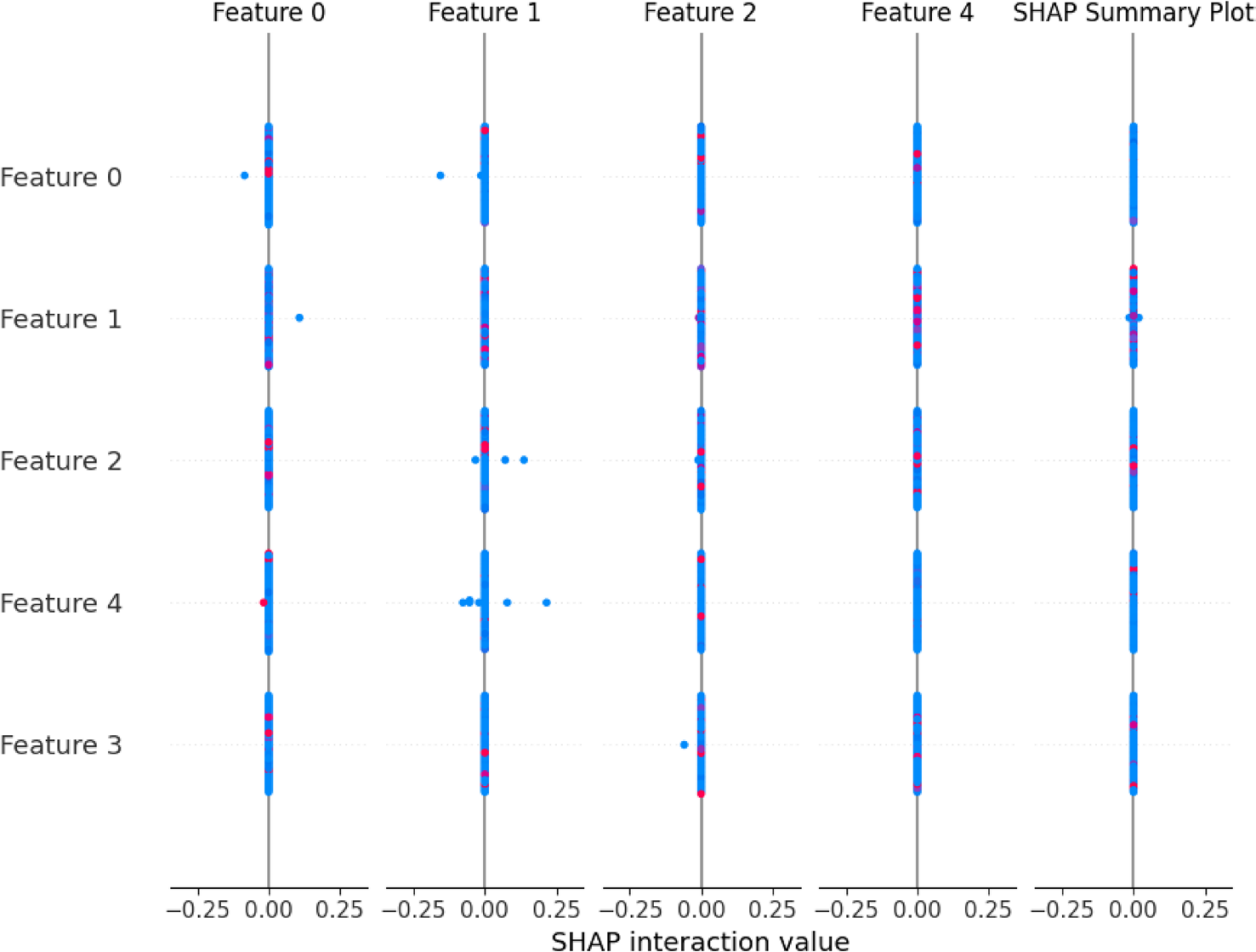
SHAP summary plot illustrating the most influential YAMNet embedding dimensions affecting the model predictions. The analysis highlights features related to high-frequency respiratory energy and temporal variations that are important for distinguishing asthma and pneumonia. The results also support the relevance of key handcrafted acoustic descriptors such as MFCC, spectral centroid, and zero-crossing rate.

YAMNet-derived embeddings were subjected to SHAP (SHapley Additive Explanations) in order to investigate the interpretability of the model’s internal decisions. The most significant latent features influencing the model’s final predictions are highlighted in the summary plot that results, which is displayed in Fig. 11. High-frequency energy and sudden temporal changes were linked to high-ranking traits, which were especially helpful in the classification of pneumonia and asthma. This interpretability layer boosts confidence in the model’s judgment.

**Fig. 12:**
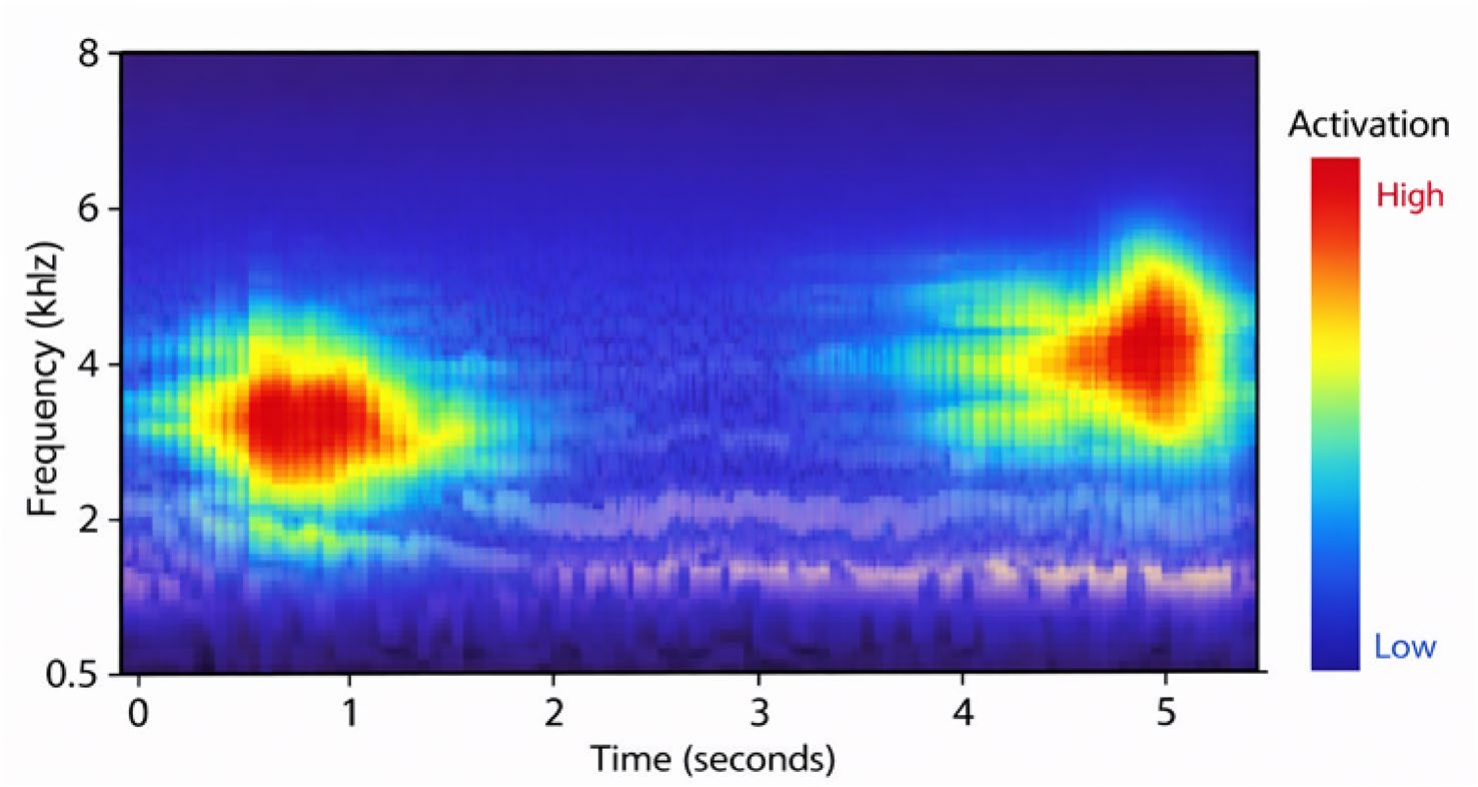
Grad-CAM visualization highlighting the most influential time–frequency regions in the mel-spectrogram. Warmer colors indicate higher activation levels corresponding to respiratory acoustic events.

To further investigate the spatial attention of the proposed model, Grad-CAM visualization was applied to the mel-spectrogram representations. Grad-CAM highlights the most influential time–frequency regions that contribute to the model’s predictions by projecting gradient-based activation maps onto the input spectrogram. As illustrated in Fig. 12, the generated heatmap emphasizes high-energy frequency bands associated with respiratory acoustic events such as wheezes and crackles. This observation confirms that the model focuses on clinically meaningful acoustic patterns rather than background noise, providing additional interpretability to support the classification results.

**Fig. 13:**
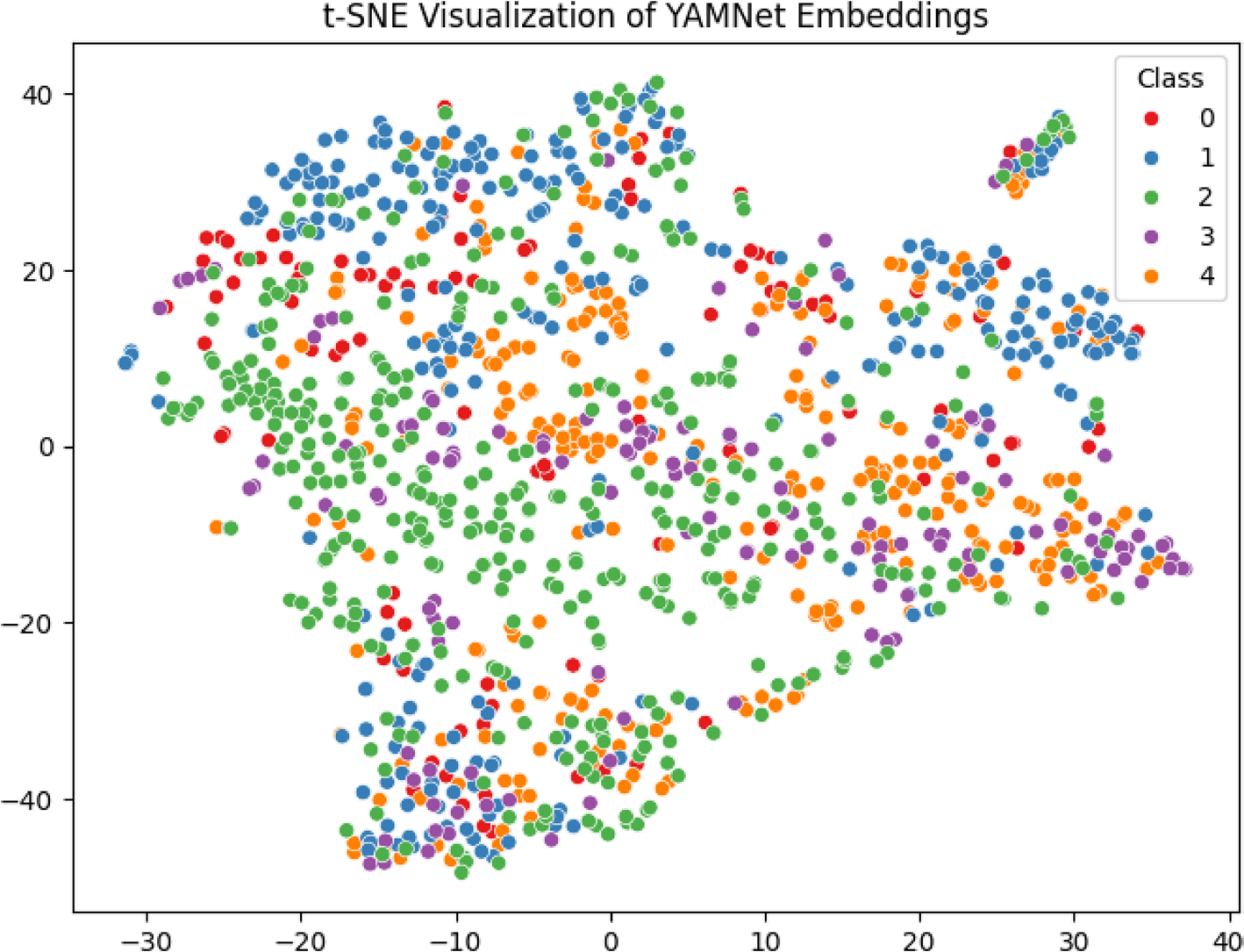
Class-wise clustering of respiratory sound samples using the t-SNE projection of the 512-dimensional YAMNet embeddings into a two-dimensional space. Healthy and pathological samples are clearly separated, while a partial overlap is observed between acoustically similar conditions such as bronchitis and asthma.

To further explore the learned feature space, the 512-dimensional YAMNet embedding is shown in Fig. 13. The visualization reveals clear class separability, with healthy samples forming a distinct cluster, while a partial overlap is observed between bronchitis and asthma. This overlap is expected due to their shared acoustic traits, such as wheezing and irregular airflow, and may also be influenced by limited sample size and variability in recording conditions. Nevertheless, the confusion matrix confirms that misclassifications are minimal and largely restricted to these acoustically similar classes, underscoring the robustness of the proposed model.Most respiratory illnesses can be clearly distinguished from one another in the resulting clusters, particularly between the healthy and sick classes. While healthy signals constituted a clear and distinct category, samples of bronchitis and asthma emerged in closely adjacent locations, demonstrating their acoustic similarities. The model’s capacity to extract semantically significant and class-discriminative representations from unprocessed audio input is further supported by this visual evidence.

**Fig. 14:**
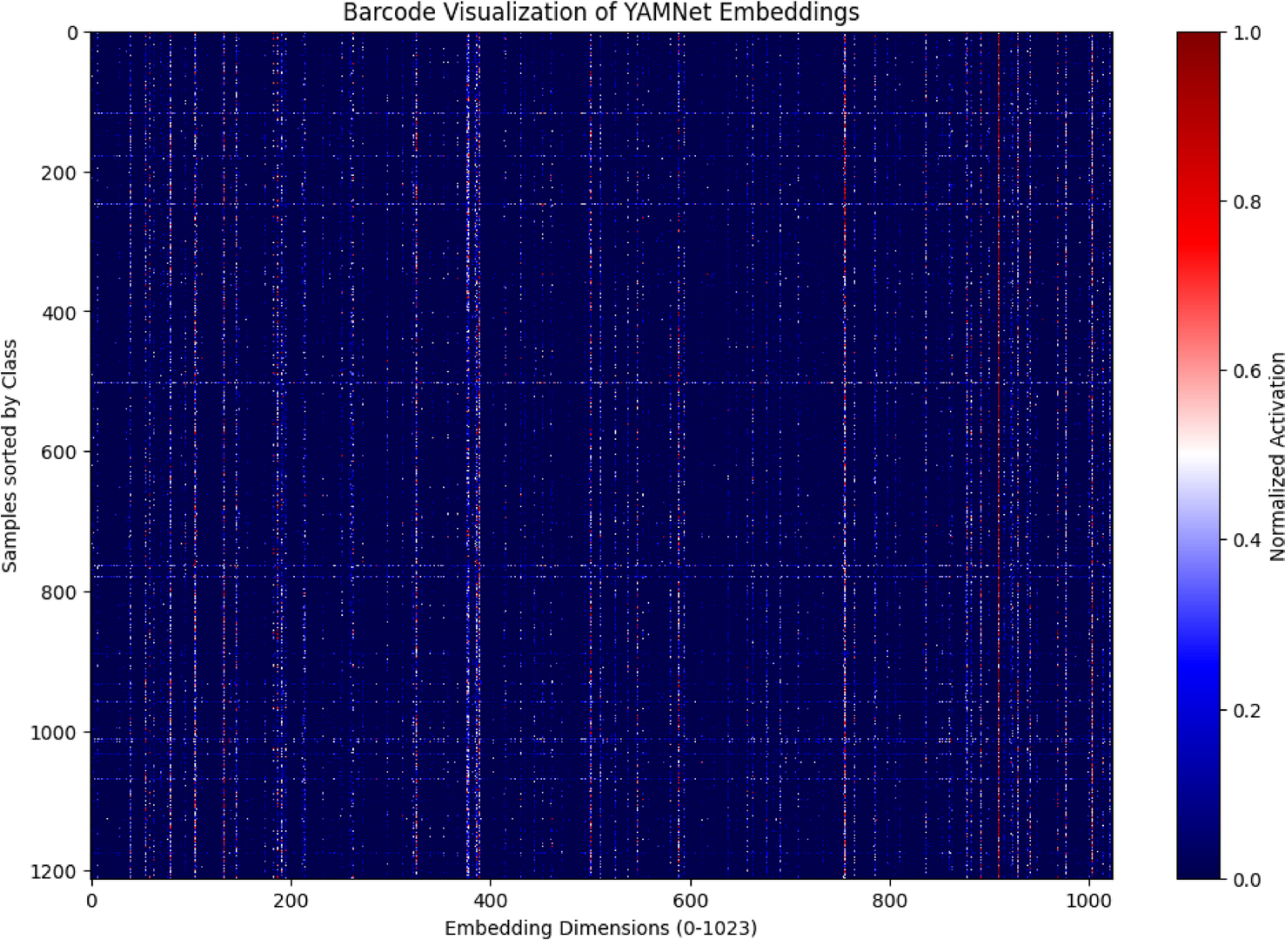
Barcode heatmap of YAMNet embeddings across respiratory audio samples: color intensity indicates normalized activation, emphasizing class-specific patterns; rows indicate individual samples; columns represent 1024 latent features.

A barcode-style heatmap was created in order to look more closely at the distribution of learnt audio representations, as seen in Fig. 14. One respiratory audio sample is represented by each row in the picture, and one of the 1024 latent embedding dimensions that YAMNet extracted is represented by each column. Normalized activation values are shown by colors, which range from low (blue) to high (red). The model’s capacity to encode class-specific auditory properties is demonstrated by the picture, which displays unique activation patterns across various breathing situations. These structured variations imply that discriminative features that are helpful for downstream classification are captured by YAMNet embeddings. As an illustration of the acoustic presence of wheeze, asthma samples typically show denser and stronger activations in higher-frequency components. Stable airflow is associated with more constant and periodic activation patterns in healthy recordings. On the other hand, samples from bronchitis and COPD show weaker or more fragmented activations, which correspond to decreased spectral diversity and airway obstruction. The model’s ability to capture clinically significant representations instead of arbitrary changes is confirmed by these condition-specific distinctions.

### Quantitative results

**Fig. 15:**
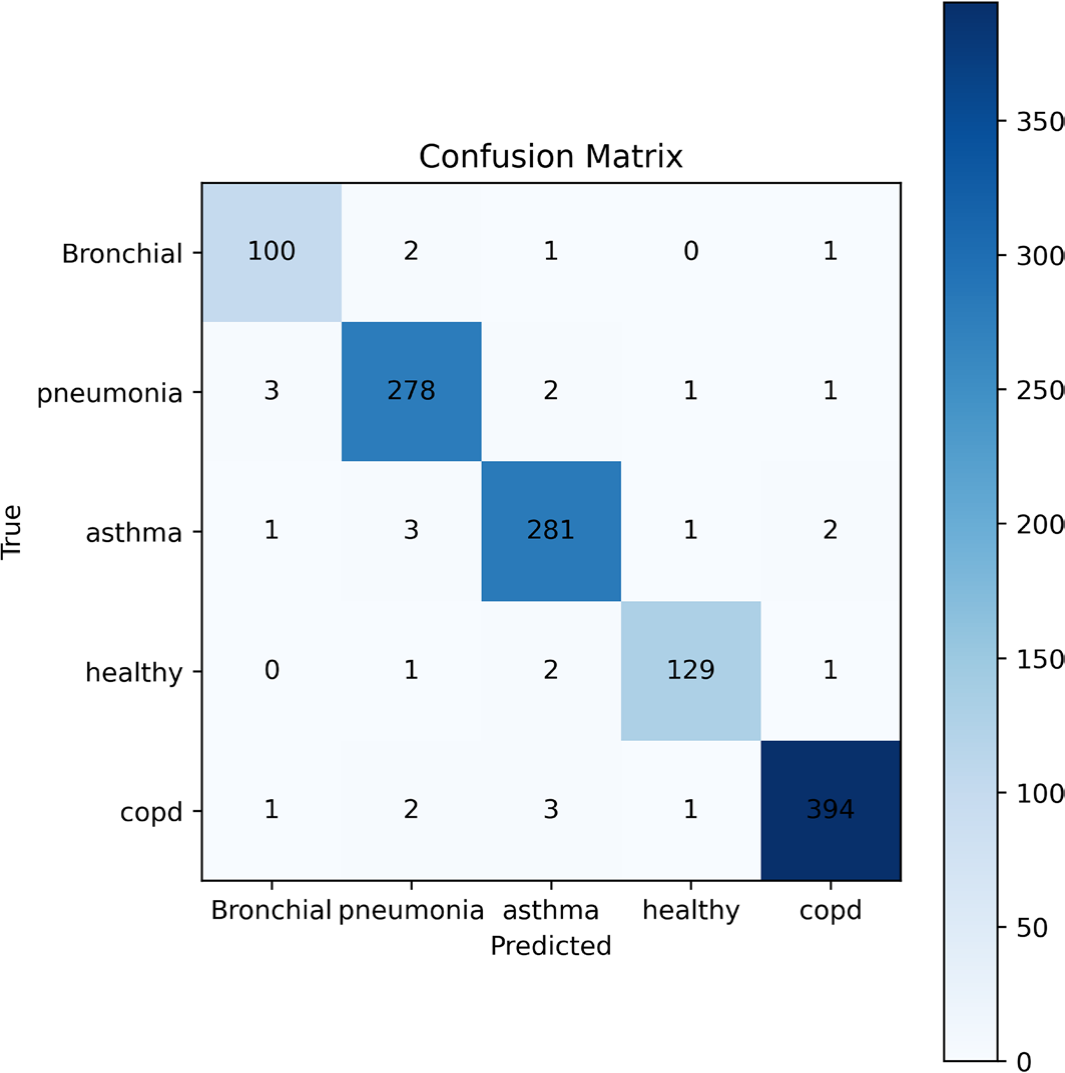
Aggregated confusion matrix of the proposed YAMNet-ASPP-SE model obtained by combining the predictions from all folds of the stratified five-fold cross-validation.

**Fig. 16:**
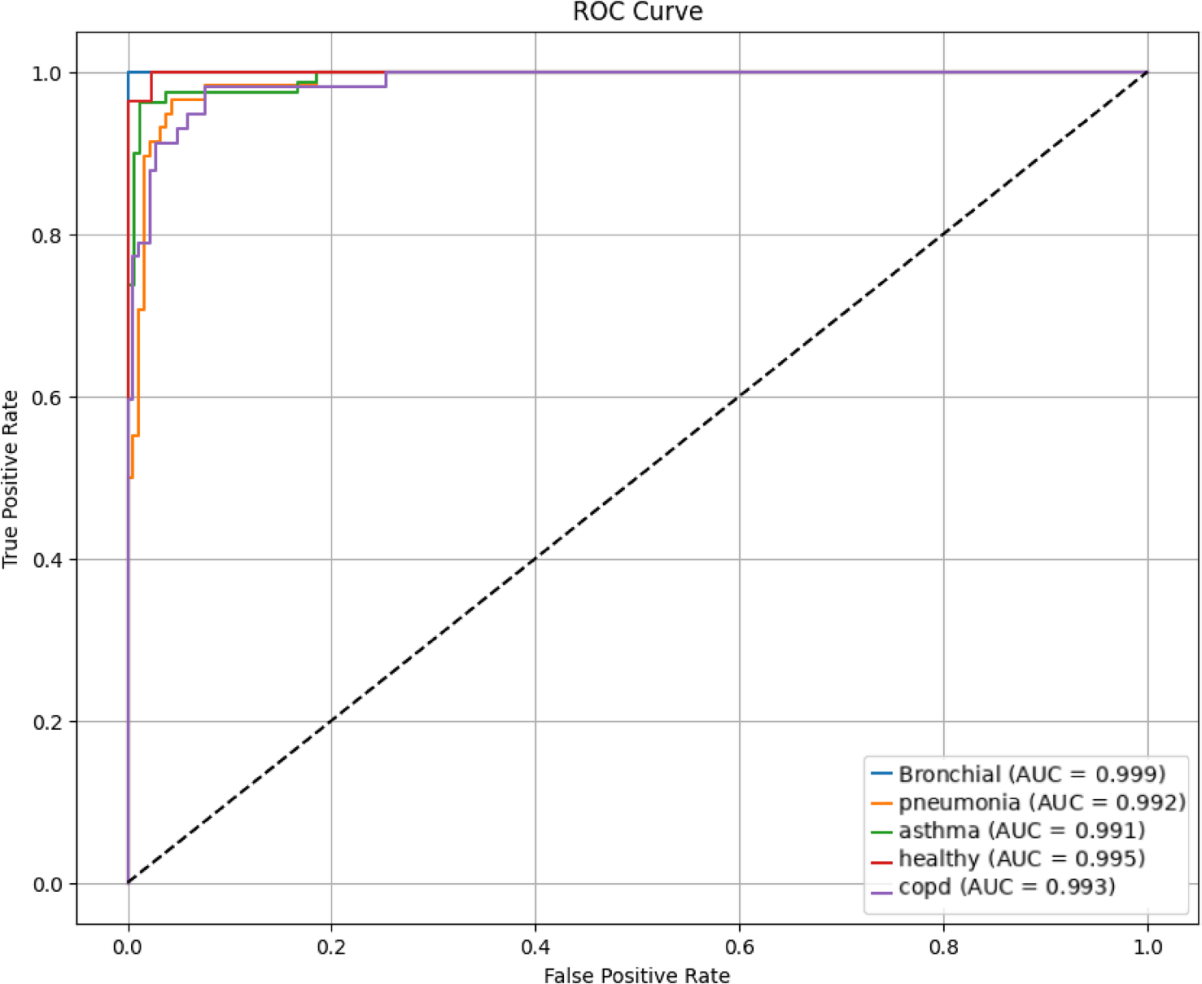
One-vs-Rest (OvR) strategy receiver operating characteristic (ROC) curves for each of the five classes. The great discriminative capacity of the model is confirmed by the area under the curve (AUC) values, which show outstanding class separability.

**Fig. 17:**
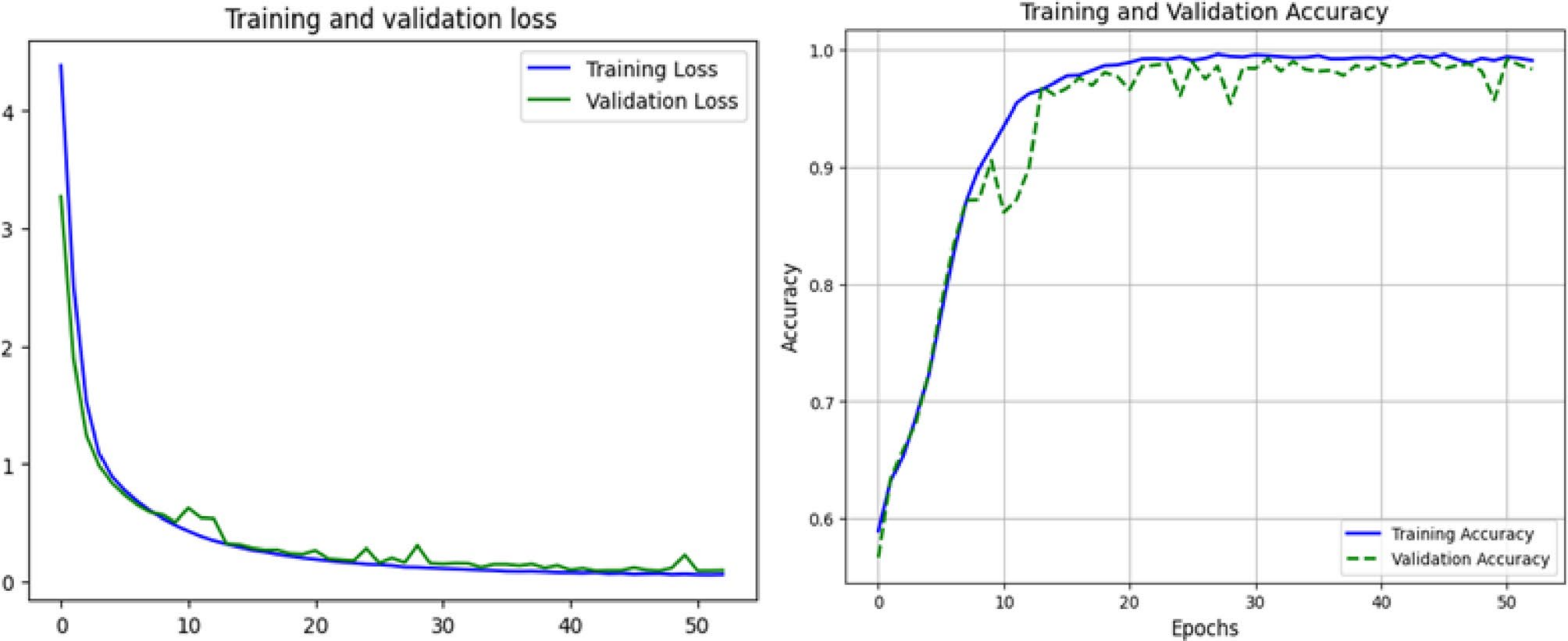
Accuracy and loss curves for training and validation over time. The model’s outstanding generalization capacity is highlighted by the consistent drop in loss and the smooth convergence of accuracy, which show stable learning dynamics, efficient optimization, and few indications of overfitting.

A stratified five-fold cross-validation approach was used to thoroughly assess the suggested deep learning architecture’s efficacy. This made sure that every class was fairly represented in both the training and validation splits. Accuracy, precision, recall, F1-score, and the macro-averaged Area Under the Receiver Operating Characteristic Curve (AUC-ROC) were among the performance indicators that were computed. To provide statistical robustness, the final performance values were presented as the mean ± standard deviation for each fold. With an average accuracy of 98.6% ± 0.14, the model showed strong overall classification performance . With an F1-score of 97.5% ± 0.14, the trade-off between sensitivity and specificity was ideal. With respective scores of 97.76% ± 0.19 and 97.42% ± 0.15, precision and recall were also good. Additionally, the model’s remarkable capacity to distinguish between the five respiratory conditions−asthma, bronchitis, COPD, pneumonia, and healthy−was highlighted by the macro-AUC score of 99.1% ± 0.14. The aggregated confusion matrix obtained by combining the predictions from all folds of the stratified five-fold cross-validation is presented in Fig. 15. It should be noted that the confusion matrix represents aggregated predictions across all folds, while the reported performance metrics are averaged across the five-fold cross-validation. Therefore, the aggregated confusion matrix does not directly correspond to the averaged performance metrics, which explains the slight difference in reported accuracy values. This clarification ensures transparency and prevents misinterpretation of the reported results. The matrix illustrates the distribution of predictions across all classes. A strong diagonal pattern indicates high classification consistency and reliable model performance. Misclassifications are relatively rare and mainly occur between acoustically similar respiratory conditions, such as pneumonia and bronchial diseases, due to overlapping temporal and spectral breathing characteristics. The ROC curves demonstrate near-perfect separation, with class-wise AUC values ranging from 0.991 to 0.999, reflecting slight but realistic deviations from perfect classification (Fig. 16). It should be noted that none of the classes achieved a perfect AUC value of 1.0. The reported AUC values range between 0.991 and 0.999, which more accurately reflect the model’s realistic classification performance. Minor visual differences between ROC curves are therefore expected and consistent with the reported AUC values . Stable learning and lack of overfitting are indicated by the smooth convergence of the training loss and the accuracy curves (Fig. 17). This speed is ascribed to the integration of ASPP and SE modules, which improve multi-scale and channel-specific feature refinement, with YAMNet embeddings for semantic feature extraction.

### Feature importance analysis

To further investigate the contribution of handcrafted acoustic features, a feature importance analysis was conducted using SHAP-based interpretation (Fig. 11). This analysis helps identify the most influential acoustic descriptors used by the model and reduces potential redundancy among handcrafted features.

The results indicate that MFCC coefficients, spectral centroid, and zero-crossing rate contribute significantly to the classification performance, as they capture important spectral and temporal characteristics of respiratory sounds. In contrast, features such as RMS energy and spectral bandwidth exhibit comparatively lower importance, suggesting that some handcrafted descriptors may contain redundant informationas summarized in Table 3.

**Table 3:** Importance ranking of handcrafted acoustic features.

Feature	Importance level
MFCC	High
Spectral centroid	High
Zero crossing rate	Medium
Chromagram	Medium
Spectral bandwidth	Low
RMS energy	Low

### Ablation study on segmentation length and data augmentation

To justify the preprocessing design choices, an ablation study was conducted to evaluate the impact of segmentation length and data augmentation on the model performance. Specifically, we investigated different segmentation durations as presented in Table 4 and compared model performance with and without the augmentation pipeline, as shown in Table 5. The results demonstrate that the proposed configuration provides the best balance between capturing complete respiratory cycles and improving model generalization.

**Table 4:** Ablation study evaluating the effect of segmentation length on model performance.

Segmentation length	Accuracy (%)	Precision (%)	Recall (%)	F1-score (%)
3 s	96.8	96.1	95.7	95.9
4 s	97.5	97.0	96.8	96.9
6 s (proposed)	98.6	97.76	97.42	97.5

**Table 5:** Ablation study evaluating the effect of data augmentation.

Configuration	Accuracy (%)	F1-score (%)	AUC (%)
Without augmentation	95.9	95.1	97.8
With augmentation (proposed)	98.6	97.5	99.1

### Comparative performance with baseline models

To examine whether reshaping the 1024-dimensional embeddings into 32 $\times$ 32 pseudo-images affects temporal dependencies, additional experiments were conducted using sequence-preserving architectures. In particular, 1D-CNN and CNN+LSTM models were implemented to process the respiratory signals directly as temporal sequences without spatial reshaping. Using the same dataset and 5-fold cross-validation technique, a thorough comparison was carried out against a number of baseline models in order to further evaluate the efficacy of the suggested YAMNet + ASPP + SE model. Performance metrics for various architectures and input types are compiled in Table 6. It should be noted that variability measures (standard deviation) are reported only for the proposed model and models evaluated within this study, while other baseline models include only the available reported metrics. This ensures a fair comparison, as standard deviation values are reported only when available from consistent cross-validation experiments. For baseline models, only the metrics reported in their original studies are presented, and missing values are indicated by (–). This reporting strategy was adopted to ensure fairness and avoid misleading comparisons when variability measures are not consistently available across prior studies. The proposed model achieved the highest overall accuracy of 98.6% ± 0.14 among the evaluated baseline models. It also demonstrated strong performance in terms of F1-score (97.5% ± 0.14), precision (97.76% ± 0.19), recall (97.42% ± 0.15), and macro-AUC (99.1% ± 0.14).

While conventional models like Decision Tree and XGBoost were only able to get accuracies of 75–84%, spectrogram-based CNN models like *EfficientNetB4* and *ResNet50* were able to attain accuracies of approximately 87–89%. The *YAMNet + MLP* configuration was the closest competitor, achieving 94.1% accuracy, although it still lacked generalization and interpretability.These results demonstrate the effectiveness of combining semantic embeddings (from YAMNet) with sophisticated modules such as SE (Squeeze-and-Excitation) and ASPP (Atrous Spatial Pyramid Pooling), which allow the model to capture both local and global acoustic patterns.

Furthermore, the reliable cross-validation outcomes support the suggested method’s generalizability and resilience. There were very slight variations in accuracy (± 0.14) and F1-score (± 0.14) across the five-fold cross-validation results, which were constant across folds. No one fold displayed a discernible decline or increase in performance, suggesting that the model behaved consistently throughout the dataset’s many partitions. This stability further supports the robustness of the suggested framework by indicating that it generalizes well and is not unduly affected by changes in the training/validation split.

The suggested YAMNet + ASPP + SE model showed good computational efficiency in addition to predictive performance. On an NVIDIA Tesla T4 GPU, training took about 10 min per fold, while spectrogram-based CNN baselines (e.g., ResNet50, EfficientNet) took more than 20 min. With an average of just a few milliseconds per sample, inference was lightweight, supporting its viability for use in near real-time applications. Although tree-based classifiers (such as XGBoost and Decision Tree) learned nearly immediately, their accuracy was significantly worse.These findings show that the significant improvements in accuracy and resilience provided by the suggested framework outweigh the slight increase in processing cost.

**Table 6:** Comparative performance of the proposed YAMNet + ASPP + SE model against baseline models using five-fold cross-validation.

Model	Input type	Accuracy (%)	Precision (%)	Recall (%)	F1-score (%)	AUC (%)	Cross-validation
CNN (EfficientNetB3)	Spectrogram (128 $\times$ 128 $\times$ 3)	87.3	85.6	84.8	85.1	86.5	Five-fold
CNN (EfficientNetB4)	Spectrogram (128 $\times$ 128 $\times$ 3)	87.7	86.8	85.5	86.0	87.4	Five-fold
CNN (EfficientNetB6)	Spectrogram (128 $\times$ 128 $\times$ 3)	88.9	83.7	83.2	83.6	85.1	Five-fold
CNN (ResNet50)	Spectrogram (128 $\times$ 128 $\times$ 3)	88.9	85.2	84.9	85.0	87.0	Five-fold
YAMNet + ASPP + SE	YAMNet + ASPP + SE	98.6 ± 0.14	97.76 ± 0.19	97.42 ± 0.15	97.5 ± 0.14	99.1 ± 0.14	Five-fold
YAMNet + MLP	YAMNet embeddings (512-D)	94.1	92.8	93.7	93.2	96.5	Five-fold
Decision tree	Tabular extracted features	75.0 ± 0.03	76.2	75.4	75.3	–	Five-fold
GBDT	Tabular extracted features	82.0 ± 0.03	82.7	81.9	82.1	–	Five-fold
XGBoost	Tabular extracted features	84.0 ± 0.03	85.0	83.7	84.3	–	Five-fold
1D-CNN + LSTM	Raw time-series audio	76.0 ± 0.02	76.8	75.9	76.1	–	Five-fold
2 $\times$ 1D-CNN	Raw time-series audio	59.0 ± 0.10	60.5	58.7	59.2	–	Five-fold

### Comparative performance with prior studies

The proposed YAMNet + ASPP + SE model is compared with several prior methods that employed similar or comparable respiratory audio datasets, as summarized in Table 7. It should be noted that some prior studies did not consistently report all evaluation metrics; therefore, only the available values are presented.

With an accuracy of 98.6% ± 0.14, the proposed model outperformed earlier studies such as Tawfik et al. (94.0%) and Ehtesham et al. (93.0%), achieving the best overall result. In addition, the proposed model surpassed more recent deep learning approaches, including Roy and Satija (95.2%) and Lee et al. (96.1%), demonstrating the highest performance among all compared methods.

Furthermore, the precision (97.76%), recall (97.42%), F1-score (97.5%), and AUC (99.1%) of the proposed model are consistently higher than the reported values in prior studies. Notably, improvements over strong baselines such as Lee et al. and Roy and Satija are particularly evident in terms of AUC and F1-score, indicating enhanced classification reliability and robustness.

To evaluate whether the observed performance improvements are statistically significant, a paired t-test was conducted between the proposed model and the best-performing baseline using results obtained from five-fold cross-validation. The resulting p-value ($p < 0.05$) confirms that the improvement achieved by the proposed model is statistically significant.

Direct comparison remains partially challenging due to differences in datasets, preprocessing pipelines, and evaluation protocols across studies. However, the integration of pretrained YAMNet embeddings with ASPP and SE modules significantly enhances feature extraction and model attention, leading to superior performance.

Unlike prior research, the proposed model maintains robustness through stratified five-fold cross-validation and provides interpretability using SHAP analysis. Additionally, while earlier works such as Tawfik et al. relied on the Asthma Detection Dataset v2 (Kaggle) with limited preprocessing, the proposed framework effectively addresses these limitations through normalization and extensive data augmentation techniques.

To improve generalization, we specifically applied normalization and extensive data augmentation techniques−such as noise injection, pitch shifting, and temporal stretching-to reduce variability and enrich the dataset. This strategy, combined with pretrained YAMNet embeddings and advanced modules like ASPP and SE, enabled the proposed model to outperform prior approaches on the same dataset, demonstrating the effectiveness of addressing data scarcity through strong preprocessing and modern deep learning architectures.

**Table 7:** Performance comparison with prior studies.

Author(s)	Accuracy (%)	Precision (%)	Recall (%)	F1 (%)	AUC (%)
Tawfik et al.^19^	94.0	92.8	93.7	88.0	97.0
Ehtesham et al.^24^	93.0	–	–	88.0	97.0
Roy and Satija^25^	95.2	94.1	94.8	94.4	98.2
Lee et al.^26^	96.1	95.5	95.2	95.3	98.5
Proposed model	98.6 ± 0.14	97.76 ± 0.19	97.42 ± 0.15	97.5 ± 0.14	99.1 ± 0.14

### Discussion

The suggested YAMNet + ASPP + SE model’s demonstrated strong performance in detecting acoustic patterns to detect minute acoustic changes linked to various respiratory disorders. The combination of multi-scale attention mechanisms and semantic embeddings allowed the model to generalize effectively across classes, even in the face of difficult circumstances like overlapping symptoms, in contrast to typical models that only use hand-crafted features or shallow architectures. Notably, the model showed resilience in differentiating between acoustically similar conditions such as bronchitis and asthma, an area where previous research has had difficulty. This illustrates the advantage of integrating the pretrained embeddings of YAMNet with the contextual diversity-capturing capabilities of the ASPP module and the adaptive feature weighting of the SE block. Furthermore, the model’s clinical reliability is strengthened by the additional interpretability provided by SHAP plots and feature space visualization using t-SNE, which provide information on decision boundaries and contributing factors. In conclusion, by attaining strong performance , enhancing transparency, and establishing scalability for practical screening applications, the suggested approach provides a competitive approach for respiratory audio categorization.

Although the proposed model achieved high performance on the Asthma Detection Dataset v2, the evaluation was conducted using stratified five-fold cross-validation to reduce the risk of overfitting and ensure robust performance estimation. Nevertheless, relying on a single dataset may limit the generalizability of the findings. Future work will therefore focus on validating the proposed framework on additional independent respiratory sound datasets, such as SPRSound, to further assess its robustness and generalization capability across different recording environments and patient populations. Although the proposed model achieved high classification performance, some misclassifications were observed during evaluation. These errors may occur when respiratory sounds share overlapping acoustic characteristics or when background noise affects the recording quality. Such cases highlight the inherent difficulty of distinguishing between acoustically similar respiratory conditions.

### Computational complexity and deployment considerations

Since the proposed framework targets mobile health applications, the computational efficiency of the pipeline was considered during model design. The preprocessing stage relies on lightweight audio descriptors such as MFCC, spectral centroid, and zero-crossing rate, which can be efficiently extracted using the Librosa library.The pretrained YAMNet model is based on the MobileNet architecture, which is designed for resource-constrained environments and reduces computational cost through depthwise separable convolutions. As a result, the model maintains moderate memory usage and low inference latency, making it suitable for real-time respiratory sound analysis and deployment in mobile health and telemedicine systems.

### Limitations

Despite the strong performance of the proposed model, several limitations should be acknowledged. The model was evaluated on a single dataset, which may limit generalizability. The dataset size is relatively small and collected in uncontrolled environments, introducing potential noise variability. Additionally, the study lacks external clinical validation, which is essential for real-world deployment. Additionally, slight class imbalance in the dataset may influence model performance despite the use of stratified cross-validation and class balancing techniques.

## Conclusion and future work

In order to diagnose asthma, this study suggested a sophisticated deep learning architecture that combines hand-crafted acoustic features with pretrained YAMNet embeddings. ASPP and SE modules are included for multi-scale and attention-driven feature refining. The system demonstrated broad generalization across diverse respiratory states and achieved high performance on the Asthma Detection Dataset v2 through extensive preprocessing, augmentation, and stratified cross-validation. Crucially, the approach becomes more transparent and reliable in clinical settings when SHAP-based interpretability and feature-space visualization are combined. In contrast to traditional CNNs based on spectrograms and tabular machine learning models, the suggested framework offers improved interpretability, computational efficiency, and competitive predictive accuracy compared to the evaluated approaches. However, there are still issues, such as the lack of external validation, the size limitations of the dataset, and the vulnerability to noise in uncontrolled settings.In addition, the current study does not include clinician-in-the-loop validation, which is important for assessing clinical trust and usability of the proposed system. Future work will therefore involve collaboration with medical experts to evaluate the clinical relevance of the extracted features and model predictions in real-world healthcare settings. In order to overcome these constraints, future studies should make use of bigger and more varied datasets, integrate multimodal clinical and environmental data, and assess practical viability in telemedicine and mobile health contexts. All things considered, this study shows how promising deep learning-based audio analysis might be as a basis for precise, comprehensible, and expandable computer-aided asthma diagnosis systems.

## Data Availability

The dataset used in this study, “Asthma Detection Dataset (Version 2)”, is publicly available on Kaggle at the following link: https://www.kaggle.com/datasets/mohammedtawfikmusaed/asthma-detection-dataset-version-2. All data used for training, validation, and evaluation were obtained from this dataset.
